# Shaped by Fire: Unravelling the Impact of Fire on Lizard Gut Microbiome

**DOI:** 10.1111/mec.70255

**Published:** 2026-01-28

**Authors:** Diana S. Vasconcelos, David James Harris, Pedro Tarroso, Catarina Simões, Catarina Rato, Xavier Santos, Raquel Xavier

**Affiliations:** ^1^ CIBIO, Centro de Investigação Em Biodiversidade e Recursos Genéticos, InBIO Laboratório Associado, Campus de Vairão Universidade do Porto Vairão Portugal; ^2^ Departamento de Biologia, Faculdade de Ciências Universidade do Porto Porto Portugal; ^3^ BIOPOLIS Program in Genomics, Biodiversity and Land Planning CIBIO Vairão Portugal; ^4^ APH Associação Portuguesa de Herpetologia Porto Portugal

**Keywords:** fire ecology, gut microbiota, metabarcoding, *Podarcis*, Portugal

## Abstract

In recent decades, wildfire regimes have changed significantly, with increases in frequency, severity and area affected, leading to major habitat alterations that may impact species ecology. While fire's role in plant ecology is well studied, its effects on animal biotic interactions remain poorly understood. In northern Portugal, where wildfires are common, the native rock‐dwelling lizard *Podarcis lusitanicus* may thrive postfire due to its preference for open rocky outcrops, which expand after fires. This suggests not only resilience but also a capacity for persistence in postfire disturbances driven by habitat preferences. However, changes in prey availability after fire induce dietary shifts in this insectivorous lizard, potentially affecting trophic interactions and, consequently, gut microbiota communities. Gut microbiota influence host fitness through effects on nutrition, immunity and behaviour; on the other hand, gut microbiota are affected by variations in diet and environment. This study assessed how fire history affects *P. lusitanicus* gut microbiota. Sampling occurred across 12 sites in northern Portugal, representing three fire histories: long‐unburned, burned in 2016 and burned in 2022. Cloacal swabs were analysed by metabarcoding the V4 region of the 16S rRNA gene. Results showed that gut bacterial composition varied with fire history, as well as with sex, body size and diet. Females had higher microbial richness despite similar diet richness between sexes. While microbiome composition shifted, predicted microbiome function remained relatively stable, indicating both resilience and ecological flexibility in fire‐prone environments. These findings enhance understanding of how lizard microbiomes respond to environmental disturbances and may help predict host and microbiota tolerance under changing fire regimes.

## 1 Introduction

Wildfires are a main driver of ecosystem reshaping worldwide (Bond et al. 2005), and in the last decades have become increasingly more severe and frequent (Stephens et al. 2014). Climate change, along with human activities, is escalating the risk and intensity of wildfires, significantly impacting ecosystems (Mansoor et al. 2022; Kelly et al. 2020; Bowman et al. 2020). Fires can have cascading effects on natural systems in various ways, affecting the biological communities directly (e.g., causing mortality; Santos et al. 2022; Jolly et al. 2022) as well as indirectly through habitat alteration/fragmentation (Driscoll et al. 2021). Habitat changes caused by the fires can generate shifts or adaptations in animal behaviour, for example by causing stress and altering the physiology and hormone levels (Hetem et al. 2014), by changing the available trophic niches (Simões et al. 2025) or aspects of the immune system (Albery et al. 2021; Beranek et al. 2023). Wild forest fires were shown to have a negative impact on soil biodiversity, particularly on arthropods (Robinson et al. 2013), due to the destruction of the vegetation structure and also the elimination of the organic layer in the upper part of the soil (Gongalsky and Persson 2013). Increases in fire frequency also significantly alter soil microbiota, with various deleterious effects, including increases in microbial plant pathogens and decreases in decomposition rates and carbon storage (Bowd et al. 2022). Such signatures of fire in soil microbiota can persist over years and even decades after the fire (Bowd et al. 2022; Nelson et al. 2022). Although information is still scarce, the animal microbiome also seems to be affected by fires, particularly in the case of amphibians. For example, studies show that the particulate matter and contaminants (e.g., heavy metals) released by fire ash can impact the skin and gut microbiome of amphibians (Hossack and Pilliod 2011; Dong et al. 2024).

The gastrointestinal tract of animals harbours a diverse community of microorganisms that play a crucial role in host biology, influencing various processes that affect fitness (Cryan and Dinan 2012; Thaiss et al. 2016). Gut microbial communities consist of microbial taxa that have co‐evolved with the host, along with a diverse array of transient microbes acquired from the environment (Shapira 2016). These microorganisms are known to maximise nutrient absorption, contribute to immune regulation, energy metabolism and pathogen defence (Rowland et al. 2018; Tong et al. 2019). Gut microbiota can impact brain development via neurochemical and immune signalling, affecting behaviour and cognition (Fields 2008; Cryan and Dinan 2012). Importantly, due to the role of gut microbiota in food digestibility, they may also play a crucial role in the adaptation of the host to new prey items (Delsuc et al. 2014; Hammer and Bowers 2015; Gomes et al. 2022). Gut microbial communities are shaped by host evolutionary history and various traits, such as sex and size (Youngblut et al. 2019; Song et al. 2020; Bunker, Arnold, and Weiss 2022). Gut microbial assemblages can be very plastic and influenced by many host external factors. Alterations to precipitation and temperature regimes, changes in habitat quality, dietary shifts and even fasting are just a few of the factors known to influence gut microbial communities (Bletz et al. 2016; Xavier et al. 2024; Fromm et al. 2024; Li et al. 2020; Fan et al. 2022).

Recently, studies demonstrated that reptiles' gut microbiome can be acquired through horizontal transmission, either from their environment or through both inter and intra‐specific interactions (Colston 2017; Vasconcelos et al. 2023). Host systematics and ecology have also emerged as key factors shaping reptile gut microbiota (Hong et al. 2011; Smith et al. 2021; Vasconcelos et al. 2023), with diet highlighted as an important driver of gut microbiome diversity (Montoya‐Ciriaco et al. 2020; Hernández et al. 2024). Although most of the knowledge on vertebrate gut microbiome is based on studies using mammals, birds, amphibians or fishes as models (e.g., de Jonge et al. 2022; Marques Silva et al. 2024; Zhou et al. 2020; Rosado et al. 2022), recent research in reptiles suggests that gut microbiota in reptiles can be influenced by the environmental and biotic changes caused by fire (Santos et al. 2022). Understanding how these fire‐induced changes shape the reptile gut microbiome is crucial for predicting how environmental disturbances, such as fire, may impact host health, ecological interactions and species resilience in changing ecosystems.

The main objective of our study was to investigate the effects of fire on the gut microbiota of the rock‐dwelling, insectivorous lizard *Podarcis lusitanicus*, in Northern Portugal. In the study region, this lizard showed higher abundances (Ferreira et al. 2016) and genetic diversity (Ferreira et al. 2019) in localities exposed to frequent fires. Additionally, a recent study demonstrated that although the prey richness of *P. lusitanicus* remains stable, diet composition changes between unburned and recently burned areas (Simões et al. 2025). To understand whether there were potentially deleterious changes in gut microbiota caused by fire, we examined gut microbial composition in lizards from sites with different fire histories. We compared the microbiota of lizards collected from areas that were burned in the previous year and 8 years prior to sampling, as well as areas that have remained unburned during that period, to investigate whether the potential signature of local fire history in the gut microbiome of lizards was still present several years after the fire. Moreover, we also tested whether putative fire effects were directly or indirectly mediated by dietary changes. In this context, we hypothesized that fire history would influence microbiota composition and diversity. We further expected accompanying shifts in predicted microbial metabolic functions, driven by changes in environmental microbial input and altered prey availability. Because lizard diet composition has been shown to vary across fire regimes (Simões et al. 2025), we predicted that dietary shifts would at least partially mediate fire‐induced differences in the gut microbiome. We also tested whether individual host traits such as sex and body size would shape gut microbiota, as these may reflect physiological, behavioural, or ontogenetic dietary differences. Lastly, we anticipated that the relative abundance of dominant bacterial taxa would shift in response to fire history, sex and body size, reflecting both environmental and host influences.

## 2 Material and Methods

### 2.1 Study Species, Study Area and Sampling

*Podarcis lusitanicus* Geniez, Sá‐Sousa, Guilliaume, Cluchier, & Crochet, 2014, is an insectivorous, diurnal, small‐sized lizard with a snout‐vent length ranging in males between 41.5 and 62.5 mm and between 40 and 60 mm in females (Carretero et al. 2015). This lizard is endemic to the northwestern Iberian Peninsula (Rato et al. 2025), a fire‐prone region, especially in northern Portugal where fire activity is forecasted to further increase (Carvalho et al. 2019; Tonini et al. 2018). This lizard has a small home range (< 500m^2^—Diego‐Rasilla and Perez‐Mellado 2003), suggesting that long‐distance dispersal is rare. Distribution of *P. lusitanicus* in northern Portugal is relatively irregular, with populations located on open natural rocky outcrops, and sometimes also inhabiting artificial stone walls around agricultural fields, preferring areas with rocks and few vegetation for thermoregulation and shelter (Diego‐Rasilla and Perez‐Mellado 2003). A study on *P. lusitanicus* in Gerês (northern Portugal) indicated that the abundance of this species increases in repeatedly burnt areas (Ferreira et al. 2016), possibly because after fires the rocky outcrops are exposed after the vegetation has been removed, creating new habitats that promote population growth of this lizard population (Ferreira et al. 2016). This may facilitate population expansion and even migration events from adjacent areas (Ferreira et al. 2019).

**FIGURE 1:**
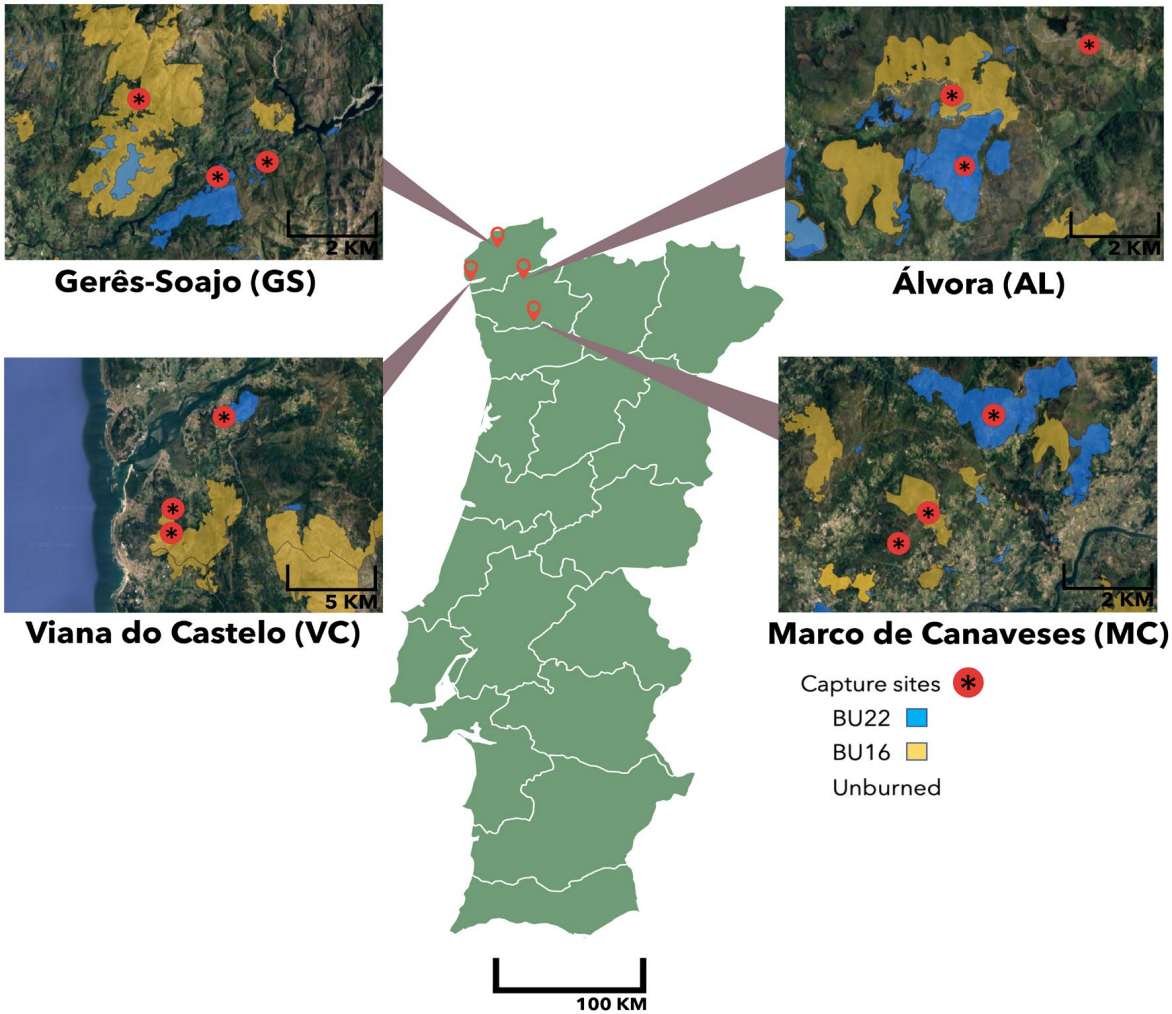
Maps of the different sampling sites: Viana do Castelo (VC), Álvora (AL), Gerês‐Soajo (GS) and Marco de Canaveses (MC)—with three capture sites selected across different historic fire conditions: Burned in 2016 (in yellow), burned in 2022 (in blue) and the long‐unburned site. Map data 2024 Google.

Between 12 May and 27 June 2023, we collected 237 lizards from 12 sites across four distinct localities in northern Portugal: Viana do Castelo (VC), Marco de Canaveses (MC), Álvora (AL) and Gerês‐Soajo (GS) (Figure 1). Within each locality, we sampled lizards in areas burned in 2022, that is, burned in the previous year to sampling (B22, *n* = 84 lizards), areas burned in 2016, that is, sampled 8 years since the last fire (B16, *n* = 77), and long‐unburned areas with no fire records between 2016 and 2022 (UB, *n* = 76). The selection of these localities was informed by the public database of burnt areas using fire records since 2015 of the Portuguese Institute for Nature Conservation and Forests (ICNF; https://geocatalogo.icnf.pt/catalogo_tema5.html).

**FIGURE 2:**
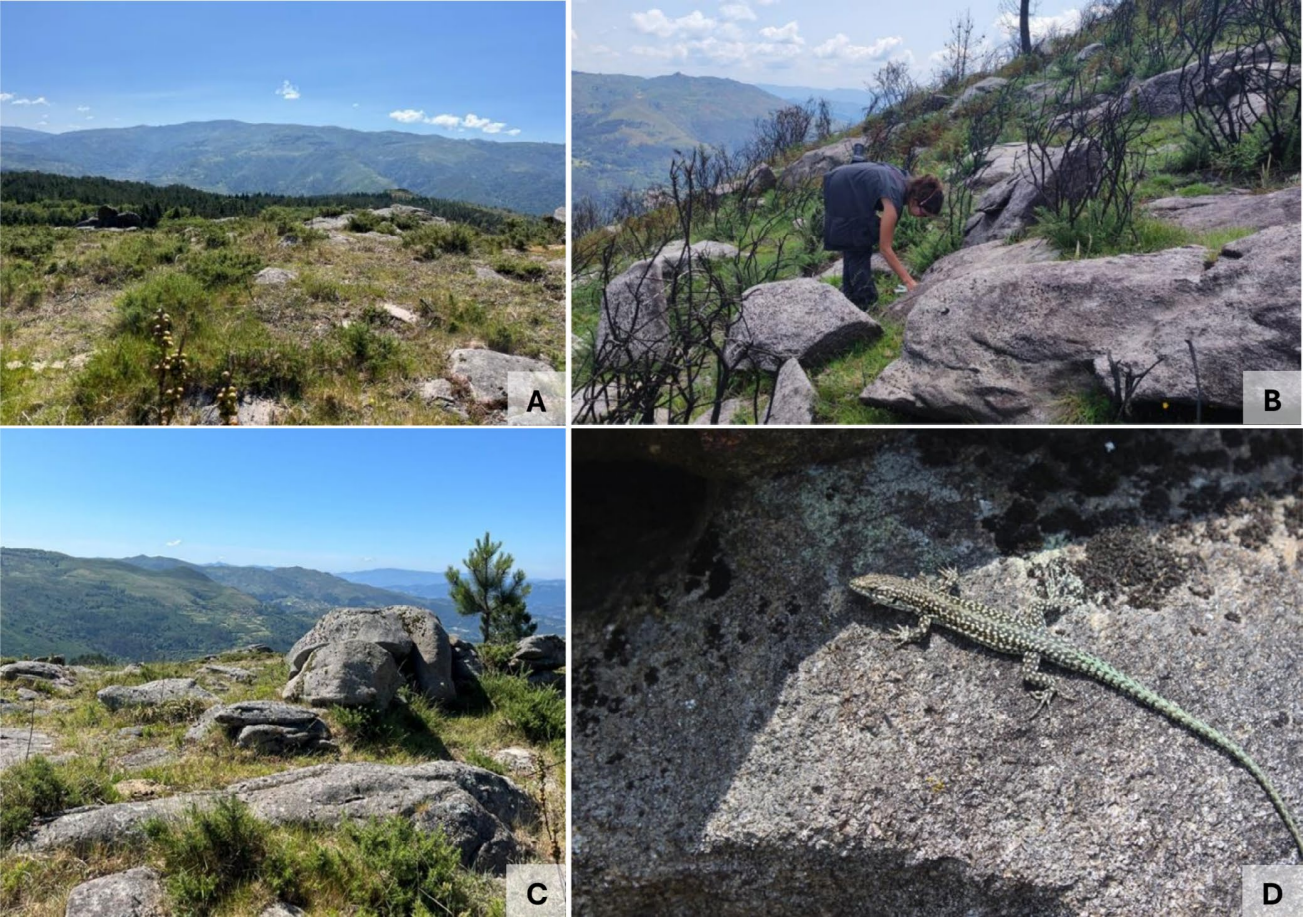
Photographs of the three different capture sites in Álvora (AL): A—area burned in 2016; B—area burned in 2022; C—long‐unburned área, and a representative male individual of *P. lusitanicus*.

Within each locality, the three areas (B16, B22 and UB) were located within a 10 km radius of each other, ensuring reduced environmental variations, and simultaneously a minimal chance for animal migration between sites. Additionally, sampling sites shared similar general habitat characteristics, such as rocky terrain and comparable vegetation structure and composition, minimising behavioural differences caused by external factors (Figure 2).

Sampling was carried out by a team of four people, starting early in the morning and finishing in the early afternoon, on sunny days with minimum temperatures in the range between 14°C and 17°C and the maximum temperatures between 22°C and 29°C. All individuals were captured using nooses. Lizards were carefully immobilised, avoiding any human contact with the cloaca. We quickly inserted a sterile cotton swab (Dryswabs, Medical Wire and Equipment, Wiltshire, UK) into the entrance of the cloaca to obtain individual microbial samples. Swabs were chosen over faecal samples since they have been described as a good proxy of microbial communities in the lower gut and cloacal tissues (Bunker, Martin, and Weiss 2022). After microbial sampling, each lizard was sexed, and the body size was measured (SVL—snout‐vent length; from snout to cloaca), using a digital calliper (±0.01 mm error). Weight was also measured with a digital scale (±0.1 g error), and each individual was photographed with the corresponding identification. Faecal pellets, if expelled during handling, were also collected and preserved in 96% ethanol to be used in a diet study (Simões et al. 2025). The swab and faecal samples were kept separately in individual tubes, stored immediately in ice boxes in the field, frozen at −20°C upon arrival at the laboratory facilities, and, at the end of the sampling season, stored at −80°C until processing. No animals died or were euthanized during sampling; all animals were released unharmed after processing at the place of collection. Experimental protocols and research were approved by the Portuguese Institute for Conservation of Nature and Forests (ICNF) (Licence 552–553/2022/CAPT).

## 3 DNA Extraction and Sequencing

In the laboratory, DNA was extracted from 237 swabs using the DNeasy PowerSoil Pro Kit (Qiagen, Hilden, Germany), according to the manufacturer's instructions, including extraction controls (*n* = 9). Individual libraries for bacterial communities, including a bacterial mock community (ZymoBIOMICS Microbial Community DNA Standard, Zymo Research, USA), extraction and PCR controls, were prepared using a standard dual indexing of the V4 region of the 16S rRNA gene (∼250 bp) with the primers 515F (5′‐GTGCCAGCMGCCGCGGTAA‐3′) and 806R (5′‐GGACTACHVGGGTWTCTAAT‐3′), following the protocol of Kozich et al. (2013). The V4 region of this gene is widely used to characterise bacterial communities in various taxa, including reptiles (e.g., Colston and Jackson 2016; Chiarello et al. 2018). The PCRs were performed as a single amplification of 35 cycles in a total volume of 50 μL with 10 μL of GoTaq Flexi colourless buffer, 5 μL of 25 mM MgCl_2_, 1 μL of each 10 mM forward and reverse primers, 1 μL of 10 mM dNTPs, 0.5 μL GoTaq DNA polymerase (Promega), 29.5 μL of PCR grade water and 2 μL of DNA template. Each PCR included a negative control using the following conditions: 95°C for 2 min, and then 35 cycles of 95°C (20 s), 55°C (15 s), 72°C (4 min) and a final elongation step of 72°C for 10 min. An initial PCR clean‐up to remove unused primers and primer dimers was performed, using a 0.85 μL ratio of the Agencourt AMPure XP beads (Beckman Coulter, Brea, CA, USA). The mock community was processed alongside the samples to assess potential PCR and sequencing artefacts.

PCR products (including 4 negative controls) were quantified using Epoch Microplate Spectrophotometer (BioTek Instruments Inc.; Winooski, VT, USA), normalised to ensure equal concentrations across all samples and pooled together. Quality control of the pool was assessed using a TapeStation 4200 High Sensitivity D1000 Assay (Agilent, Santa Clara, CA), followed by an additional purification step using a 0.8 μL ratio of magnetic beads. A second test for quality control in the TapeStation was performed to confirm the success of the cleaning. The final pool was then sent to GENEWIZ Next Generation Sequencing laboratory for sequencing on an Illumina MiSeq sequencer using a 2 × 250 bp paired‐end (PE) configuration with PhiX (≤ 20%) added to enhance sequencing diversity. Raw sequence reads were deposited into NCBI's Short Read Archive under project PRJNA1391492.

Diet data was already available from a subset of 183 lizards and was assessed using metabarcoding of a portion of the Cytochrome Oxidase I, as described in Simões et al. (2025).

### 3.1 Sequence Denoising

Raw FASTQ files were denoised using the DADA2 pipeline (Callahan et al. 2016). After an assessment of read quality plots, the parameters for trimming and filtering were set as: trimLeft = 20, truncLen = c(220, 150), maxN = 0, maxEE = c(2, 2), truncQ = 2. The SILVA 138 database (Pruesse et al. 2007; Quast et al. 2012) was chosen for taxonomic assignment. After quality control and taxonomic assignment, sequences identified as Mitochondria and Chloroplast were removed from the dataset. To assess and control for contamination, ASVs present in extraction controls (*n* = 9) and PCR controls (*n* = 4) were removed from the dataset. The observed composition and relative abundances of the mock community closely matched the manufacturer's specifications (see Figure S1). An amplicon sequence variant (ASV) frequency table was constructed using the R package *phyloseq* (McMurdie and Holmes 2013). An approximate‐maximum‐likelihood midpoint rooted phylogenetic tree was estimated using the software QIIME2 (Bolyen et al. 2019). Normalised read counts were obtained using the negative binomial distribution implemented in DESeq2 (Love, Huber, and Anders 2014; McMurdie and Holmes 2014). The composition and abundance of taxa in the mock community were similar to those described by the manufacturer. All the following analyses were performed using the R Software v.4.3.3 (R Core Team 2024).

### 3.2 Statistical Analysis

From the matrix of ASV abundances on the gut content of each lizard, bacterial alpha‐diversity (within‐sample diversity) and community structure (beta‐diversity, based on dissimilarity between pairs of samples) were calculated using the *phyloseq* and the *picante* packages (McMurdie and Holmes 2013; Kembel et al. 2010) (see R script provided as Supporting Information). Metrics of alpha‐diversity included ASV richness, the Shannon index and Faith's Phylogenetic Diversity (PD). The ASV richness represents the number of unique amplicon sequence variants in each sample, providing a simple count of taxa (Callahan et al. 2017). The Shannon index accounts for both ASV richness and evenness, capturing diversity by considering the proportion of each taxon (Legendre and Legendre 2012). Faith's PD measures diversity as the total branch length of the phylogenetic tree connecting all taxa present in a sample, incorporating evolutionary relationships among organisms (Faith 1992). Beta‐diversity was measured using the Bray–Curtis and weighted and unweighted UniFrac phylogenetic distances between pairs of samples. The Bray–Curtis distance quantifies compositional dissimilarity based on the abundance of shared taxa, without considering phylogenetic relationships (Bray and Curtis 1957). Weighted UniFrac accounts for both the relative abundance of taxa and their evolutionary relationships (Lozupone et al. 2007), while unweighted UniFrac considers only presence or absence, making it more sensitive to differences in rare lineages (Lozupone and Knight 2005). Principal coordinate analysis (PCoA) was used to visually assess dissimilarity among groups. Firstly, we tested the effect of locality on both alpha and beta diversity metrics with no significant effects found. We therefore modelled gut microbiota alpha‐diversity using Generalised Linear Mixed Effects Models (GLMM), with fire regime (B16, B22, UN), SVL, and sex as fixed effects, and locality as a random effect (lmer(alpha‐diversity ~ fire regime + SVL + sex + (1|locality))). Similarly, for beta diversity, we used permutational analysis of variance (PERMANOVA) with 9999 permutations in the *adonis2* function from the *vegan* R package (Oksanen et al. 2013), including fire regime, SVL and sex as predictors and stratifying permutations by locality to account for the sampling design using the formula: (adonis(beta‐diversity ~ fire regime + SVL + sex, strata = Locality)). Pairwise PERMANOVA comparisons were also stratified by locality and conducted to assess differences in microbiome composition between groups. FDR‐adjusted *p*‐values were used to correct for multiple comparisons and reduce the likelihood of false positives.

Additionally, given the results of Simões et al. (2025), which reported significant compositional dietary differences between recently burned (B22) and long‐unburned sites (UN), we used the available diet data from a subset of individuals (*n* = 183) to test whether these dietary shifts were mediating the observed changes in gut microbial composition. To perform this analysis, we converted the beta‐diversity matrices for that subset of individuals for both diet and microbiome (Jaccard and Bray–Curtis, respectively) into pairwise distance vectors, where each value represented the dissimilarity between a pair of individuals. Differences in fire regimes between each pair of sites were captured by a recoded fire regime variable, assigned to one of four categories: same fire‐history site, B16 vs. B22, B16 vs. UN and B22 vs. UN. Sex was coded as either the same or different between sample pairs, and body size differences were included as Euclidean distances (Table S1). Given our sampling design, the limited body size variation among adult lizards and the absence of a diet–body size relationship (Simões et al. 2025), interaction terms were not included in the statistical models.

We implemented linear mixed‐effects models using the *nlme* R package (Pinheiro et al. 2021) on both the full dataset (all individuals with microbiome data) and for the dataset containing only the subset of individuals with available diet data. Linear regression models by maximum likelihood (ML) were fitted to allow for AIC‐based model comparison. The full model was specified as BetaMicrobiome ~ BetaJaccDiet + FireRegimedistance + SVLdistance + SEXdistance, and the diet term was excluded when the full dataset was used. Full models were compared against a null model (BetaMicrobiome ~1).

Differences in the relative abundance of the most represented taxa at the phyla and genera levels (those with relative abundance ≥ 3%) were also assessed using a GLMM with fire regime, sex and SVL as explanatory variables (lmer(bacterial taxa ~ fire regime + sex + SVL + (1|locality))). A total of 7 phyla and 19 genera met the ≥ 3% relative abundance threshold and were included in the comparisons. Significative associations with fire regime and sex were explored using boxplots, while associations with body size (SVL) were visualised using a scatter plot and a linear regression implemented in *ggplot2* (Wickham 2016).

To test whether there were additional, albeit less abundant taxa associated with areas that were burned in different fire‐regimes, a differential abundance analysis using the entire dataset was also performed using the R package *Corncob* (v 0.1.0) (Martin et al. 2020). *Corncob* fits beta‐binomial regression models that account for overdispersion and compositionality, allowing simultaneous testing of changes in both mean abundance and variability. We conducted pairwise comparisons between unburned areas and those burned in 2016 or 2022 by subsetting the dataset accordingly and modeling ‘Fire regime’ as a fixed effect in both the abundance and dispersion components. Wald tests were used to identify differentially abundant taxa at a false discovery rate (FDR) threshold of 0.05 (Benjamini and Hochberg 1995).

Predicted bacterial metabolic functions were estimated using the Phylogenetic Investigation of Communities by Reconstruction of Unobserved States software (*PICRUSt2*), using the default weighted nearest sequenced taxon index (NSTI) cutoff (Bolyen et al. 2019; Douglas et al. 2019). Predicted functions were collapsed using the Kyoto Encyclopedia of Genes and Genomes (KEGG) pathway metadata (Kanehisa et al. 2019). *DESeq2* differential abundance test on predicted KEGG Orthology (KO) counts was implemented in *ggpicrust2* R package (Yang et al. 2023) was used to test for differences in predicted metabolic function between samples collected from sites burned in 2016, 2022 and unburned.

## 4 Results

**FIGURE 3:**
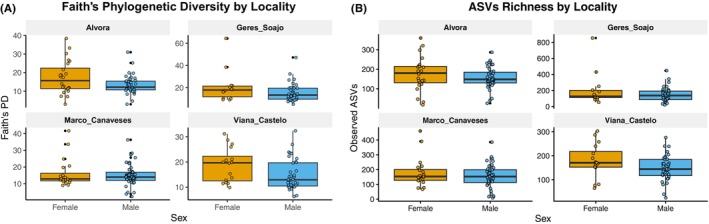
Boxplots showing alpha diversity metrics across localities in *P. lusitanicus* . (A) Faith's Phylogenetic Diversity (PD) and (B) ASV Richness are displayed by sex (Female and Male). Points represent individual samples to highlight data distribution within each sex category.

After filtering, the final ASV table encompassed 9122 unique ASVs, which included a total of 36 bacterial phyla. The most abundant phyla were Firmicutes (45.25%), Bacteroidota (24.40%), Actinobacteroidota (12.78%), Proteobacteroidota (9.17%) and Campylobacterota (3.34%) (Table S2). Among the three alpha diversity metrics assessed, only sex had a significant effect on gut microbial diversity, with females exhibiting higher diversity than males in both Faith's phylogenetic diversity and observed ASV richness. Shannon diversity did not show significant differences across fire regimes, SVL, or sex (Table 1, Figure 3).

**TABLE 1:** Results of linear mixed‐effects models testing the effects of fire regime (Fire_regime), sex, and snout‐vent length (SVL) on alpha diversity metrics (Shannon diversity, Faith's Phylogenetic Diversity [PD] and Observed ASVs).

Diversity metric	Factor	Level (vs. reference)	Coefficient	*p*
Shannon	Fire_regime	fire_2016 (ref)	—	—
		fire_2022	0.087	0.593
		unburned	0.090	0.585
	Sex	Male (vs. Female)	0.132	0.359
	SVL	—	−0.002	0.871
PD	Fire_regime	fire_2016 (ref)	—	—
		fire_2022	0.846	0.493
		unburned	−0.577	0.650
	Sex	Male (vs. Female)	**−3.131**	**0.005**
	SVL	—	−0.066	0.507
ASVs richness	Fire_regime	unburned (ref)	—	—
		fire_2016	17.019	0.242
		fire_2022	23.800	0.100
	Sex	Male (vs. Female)	**−28.879**	**0.024**
	SVL	—	−0.394	0.726

*Note:* Locality was included as a random effect in all models. Model coefficients (slopes) indicating the direction and magnitude of each predictor's effect relative to the reference level are depicted, along with corresponding *p*‐values. Statistically significant results are highlighted in bold.

**FIGURE 4:**
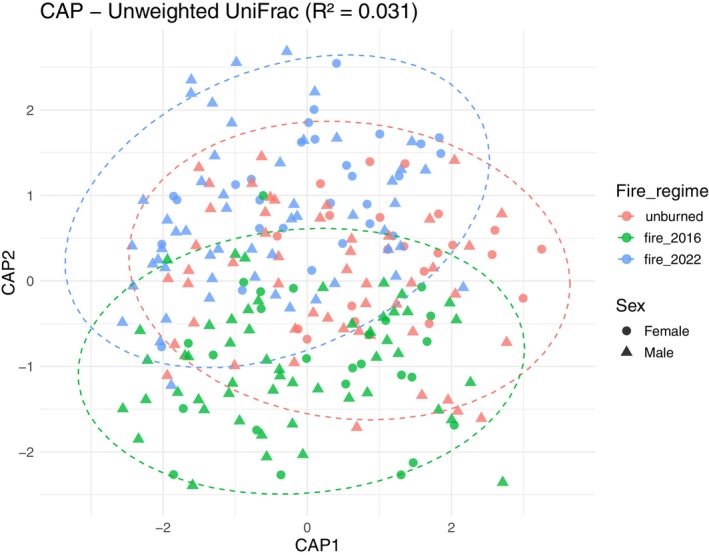
Canonical Analysis of Principal Coordinates (CAP) ordinations of gut microbiome composition based on unweighted UniFrac dissimilarity. Points represent individual samples, coloured by fire history (Fire_regime) and shaped by sex. Ellipses indicate 95% confidence intervals for each fire history group. Title show the proportion of variance (*R* ^2^) explained by the constrained model (Type_fire, Sex, SVL).

The PERMANOVA analyses showed significant differences in microbial composition between lizards captured in sites with different fire regimes using unweighted UNIFRAC and Bray‐Curtis distances. Significant pairwise differences were found between all fire regimes using both Unweighted UniFrac (adjusted *p* = 0.004–0.017; *R* ^2^ = 0.0105–0.0124) and Bray‐Curtis distance (adjusted *p* = 0.011–0.045; *R* ^2^ = 0.011–0.014) (Figure 4, Table S3 and Figure S2).

Additionally, results from the GLM model using the reduced dataset indicated that diet only had an effect on microbial composition when weighted unifrac distance was used, with results for the remaining factors similar to those of PERMANOVA encompassing the entire microbiome dataset. Using the AIC criteria, the full model outperformed the null model (all values reported in Table 2). Across all distance metrics, both PERMANOVA and generalised linear model (GLM) models applied to the full dataset identified fire regime, sex and SVL as consistent predictors of variation in gut microbial community structure, with the effect of sex being always significant.

**TABLE 2:** Results from generalised linear models (GLMs) evaluating the effects of fire regime (TypeFireVec), body size (SVLvec), sex (SEXvec) and diet (BetaJaccDiet; only in the diet subset) on gut microbial beta‐diversity in *P. lusitanicus* .

Distance metric	Dataset	L.Ratio	AIC (full/null)	Predictor	*F*‐value	*p*
Bray–Curtis	Full (no diet)	109.90	−24165.38/−24055.49	**TypeFireVec**	**36.5**	**< 0.0001**
				**SVLvec**	**8.4**	**0.0037**
				**SEXvec**	**31.0**	**< 0.0001**
	Diet subset	67.21	−17571.91/−17512.70	BetaJaccDiet	0.4	0.525
				**TypeFireVec**	**30.6**	**< 0.0001**
				**SVLvec**	**13.2**	**0.0003**
				**SEXvec**	**23.1**	**< 0.0001**
Unweighted UniFrac phylogenetic	Full (no diet)	179.53	−30015.65/−29836.12	**TypeFireVec**	**20.6**	**< 0.0001**
				SVLvec	0.09	0.766
				**SEXvec**	**133.2**	**< 0.0001**
	Diet subset	170.48	−23255.82/−23093.34	BetaJaccDiet	0.2	0.692
				**TypeFireVec**	**15.3**	**0.0001**
				SVLvec	0.6	0.427
				**SEXvec**	**155.3**	**< 0.0001**
Weighted UniFrac phylogenetic	Full (no diet)	112.44	−95174.44/−95061.99	TypeFireVec	0.15	0.697
				SVLvec	0.76	0.383
				**SEXvec**	**106.4**	**< 0.0001**
	Diet subset	132.11	−72155.77/−72031.66	**BetaJaccDiet**	**59.6**	**< 0.0001**
				TypeFireVec	1.17	0.279
				SVLvec	0.37	0.545
				**SEXvec**	**71.5**	**< 0.0001**

*Note:* Models were run using three distance metrics: Bray–Curtis, unweighted UniFrac and weighted UniFrac. Each model reports the likelihood ratio (L.Ratio), AIC values (full vs. null) and *F*‐tests for individual predictors. Analyses on the full dataset (excluding diet) were compared with a reduced subset including diet. Significant predictors (*p* < 0.05) are shown in bold.

Differences in the relative abundance of several bacterial taxa were associated with fire history, sex and body size in *P. lusitanicus* . Fire regime significantly influenced the abundance of the genus *Anaerosporobacter* (*F* = 6.16, DF = 2, *p* = 0.002), with the highest levels observed in recently burned sites (BU22) and the lowest in unburned areas (UN). Sex also affected the relative abundance of key phyla and genera. At the phylum level, females exhibited higher proportions of Actinobacteriota (*F* = 6.37, DF = 1, *p* = 0.01) and Desulfobacterota (*F* = 9.99, DF = 1, *p* = 0.001) than males (see Figure S3). Among the most represented genera, females showed higher relative abundances of an unclassified *Corynebacteriales* genus (*F* = 5.59, DF = 1, *p* = 0.01), *Parabacteroides* (*F* = 4.56, DF = 1, *p* = 0.03) and *Coprobacillus* (*F* = 4.78, DF = 1, *p* = 0.02). Finally, body size (SVL) was positively associated with *Parabacteroides* abundance across all individuals (*F* = 4.38, DF = 1, *p* = 0.03), suggesting that larger individuals tend to harbour higher levels of this genus (see Figure S4).

**FIGURE 5:**
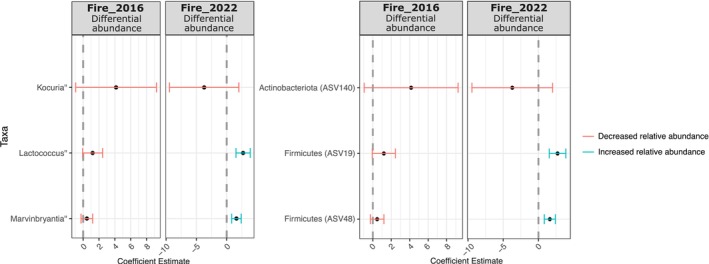
Differential abundance analysis of microbial taxa. The left panel represents significant changes at the genus level, while the right panel shows changes at the phylum level, comparing microbial communities between fire regimes in 2016 and 2022. Coloured error bars indicate significant differences, with red representing a decrease and blue representing an increase in relative abundance.

*Corncob* analysis revealed significant differential abundances in microbial taxa between areas that were burned in different years relative to unburned areas, with *Lactococcus* and *Marvinbryantia* increasing in areas that burnt in 2022, while *Kocuria* declined; at phylum level, Firmicutes (ASV19, ASV48) exhibited significant changes in areas burned in 2022, while Actinobacteriota (ASV140) stayed relatively stable (Figure 5).

Regarding the *PICRUSt2* results, fire regime produced no significant changes to the predicted metabolic function of lizard gut microbiota (all corrected *p* > 0.05, results not shown). However, these predictions are based on marker gene sequencing profiles for reference genomes and may not reflect accurate functionality of the gut microbiota of lizards; as such, these results should be interpreted as preliminary indications of potential functional stability.

## 5 Discussion

In this study, we characterised the gut bacterial microbiota of *Podarcis lusitanicus* from localities that had different fire histories in northern Portugal. The results show that microbial alpha‐diversity did not change significantly between lizards from sites subject to distinct fire regimes, but showed significant differences between sex, with females displaying higher gut microbial diversity than males. Notably, fire history had a significant effect on lizard gut microbial composition. Nonetheless, most of the microbial variation found remained unexplained by the factors considered in the analyses (diet, fire, sex and length). Although we selected our sampling sites based on similar characteristics (i.e., habitat structure, elevation, ground lithology and climatic conditions), these results indicate that other variables may have influenced our results. High microbiome variability is expected in wild animals (e.g., Xavier et al. 2020), including lizards (Lemieux‐Labonté et al. 2022), and this unexplained variation could be linked to other factors known to influence host microbiota, such as parasitism, disease, social behaviour or host age (O'Keeffe et al. 2021; Baldo et al. 2018; Rosado et al. 2023).

### 5.1 Effects of Fire and Diet in Reptile Gut Microbiota

Our findings show that fire regime significantly influences the gut microbial composition of *P. lusitanicus* , as demonstrated by both Bray–Curtis dissimilarity and Unweighted UniFrac distance, supporting our hypothesis that fire history shapes host‐associated microbial communities. Differences were evident both in metrics capturing microbial presence/absence and in those reflecting relative abundance. These impacts on gut microbiota can probably be due to changes in habitat and diet. Such patterns are consistent with those reported in previous studies showing effects of fire on microbial communities caused by changes in habitat, diet and ecological interactions (Certini 2005; Pressler et al. 2019). Although lizards can likely escape acute heat stress caused by wildfires by sheltering in burrows or rocky refuges, fires can lead to broader changes in habitat structure, climate, vegetation and prey availability, all of which may shape microbial colonisation. For example, Santos et al. (2016) documented positive short‐term responses of Mediterranean geckos to unplanned fires, suggesting some resilience or adaptation to post‐fire environments. Meanwhile, other studies have reported direct mortality of reptiles induced by forest fires and long‐term impacts on their populations (Smith et al. 2012).

As observed in previous studies on vertebrates, habitat changes can lead to differences in the composition and diversity of the gut microbiome (e.g., Amato et al. 2013; Pereira et al. 2023), including in reptiles (Vasconcelos et al. 2023). For example, it was hypothesized that *Podarcis siculus* may have recruited a high proportion of local microbial taxa into their gut following its introduction in Lisbon (Portugal) (Vasconcelos et al. 2023). Here, changes in habitat directly caused by fires may have influenced gut microbial colonisation, as supported by the observed differences in microbial community composition. Consistently, dietary analysis of the same lizard populations showed that fire history was a significant predictor of prey item composition (Simões et al. 2025). For instance, ants and spiders were seemingly absent in areas recently burned in 2022, while present in unburnt plots. Despite these differences, and much like the microbiome data presented here, lizards seem to have maintained similar prey richness across sites with different fire histories, possibly due to their generalistic diet (Arnold 1987; Sagonas et al. 2014), and the resilience or rapid recolonisation of certain arthropods in burned land plots. Arthropod responses to fire can vary widely (Swengel 2001; Moretti et al. 2004), often involving shifts in community composition, from forest specialists in unburned areas to open‐habitat specialists in burned sites (Underwood and Quinn 2010; Buddle et al. 2006), while some generalist species may persist across conditions (Swengel 2001).

Changes in available macronutrients are known to affect vertebrates' intestinal microbiota. For example, the seasonal shifts in nutrient intake led to the reconfiguration of the gut microbiota in herbivorous primates (Li et al. 2023). Differences in protein sources also alter gut microbiota in mice, affecting microbiome metabolic function as well (e.g., changes in amino acid metabolism) (Blakeley‐Ruiz et al. 2025). Similar patterns found between microbial and diet composition in the analysed lizards, that is, changes in microbial and diet composition likely elicited by different fire histories but unaltered microbial and diet richness, led us to hypothesize that diet quality could have mediated the observed changes in gut microbial community structure. However, the present results show that diet and microbial composition in the analysed lizard populations are mostly independent, suggesting that additional factors beyond diet may be driving microbial shifts. Considering that sampling took place during the species' breeding season, physiological stress may be a contributing factor. For instance, male lacertid lizards, including *Darevskia* spp. and *Lacerta agilis*, are known to experience elevated stress levels during reproduction, as evidenced by increased parasite load and immune investment (Arakelyan et al. 2019; Olsson et al. 2000), which may in turn influence gut microbiota independently of diet. These findings align with the observed differences in microbial composition between sexes, reinforcing the idea that internal host factors can play a significant role in shaping gut microbial communities during this reproductive period.

Furthermore, the abundance of Firmicutes increased in lizards from recently burned areas, a pattern that is frequently observed in environmental samples. For example, a study carried out in Mediterranean‐type ecosystems reported a notable increase in Firmicutes in burnt soils, highlighting their adaptability to such disturbed conditions (Dalias et al. 2024). Similarly, research examining prescribed burns in semi‐arid Mediterranean forests found high levels of Firmicutes soon after exposure to fire (Soria et al. 2023). This pattern is generally attributed to the ability of these bacteria to resist environmental stress and to rapidly colonise and exploit new ecological niches available in post‐fire environments. *Lactococcus* is a Firmicute that has been identified as being abundant in the guts of lizards (e.g., *Podarcis muralis* and *Anguis fragilis* ) (Lazarkevich et al. 2024), especially those with frugivorous diets (Wang et al. 2024). However, in snakes, *Lactococcus* has been identified as a keystone taxon in species that feed primarily on vertebrate prey, highlighting the influence of dietary specialisation on gut microbial composition (Zhu et al. 2025). *Lactococcus* is typically viewed as benefiting animal gut health due to their reported anti‐inflammatory properties, direct competition with pathogenic bacteria and immune system stimulation or the inhibition of the activity of toxic substances (Deng et al. 2022). In the present study, although not highly abundant, *Lactococcus* was found to be more abundant in lizards from recently burned areas, which could reflect an adaptation to changing prey items. Besides *Lactococcus*, there were three other bacterial genera (*Kocuria*, *Anaerosporobacter* and *Marvinbryantia*) that changed abundances in lizards from recently burned areas.

Fire can cause drastic fluctuations in temperature and significantly alter soil properties, which can have a negative impact on microbial survival and function (Dooley and Treseder 2012). Post‐fire changes in nutrient availability*—*such as reductions in organic carbon and nitrogen*—*can affect bacterial abundance and create less favourable conditions for *Kocuria*'s metabolic needs (Ferrenberg et al. 2013). These factors probably contribute to the reduced abundance of *Kocuria* in recently burnt environments. Although *Kocuria* is primarily known as a soil‐associated genus, its presence in the gut microbiome of various vertebrates, including reptiles, has been documented, although it is not typically dominant (Hoffbeck et al. 2024). The lower abundance of *Kocuria* observed in recently burned sites may reflect shifts in environmental exposure or indirect effects of habitat changes on microbial acquisition.

Nevertheless, our analysis indicates functional redundancy despite changes in microbiome community structure. Microbial plasticity and functional redundancy are viewed as intrinsic to the gut microbiome and a mechanism to prevent gut dysfunctionality that could lead to the onset of diseases (Celi et al. 2017). These results suggest that gut health was maintained in lizards 1 year post‐fire, despite the diet changes caused by fires, which have not elicited imbalances in the metabolic function of gut microbiota.

### 5.2 Host Factors Affect the Gut Microbiota of *Podarcis*

Differences in the microbiome composition may not exclusively be a reflection of distinct diet compositions, since gut microbes also respond to host intrinsic factors, such as physiological and immunological stressors. In our study, female *P. lusitanicus* exhibited significantly higher microbial richness and phylogenetic diversity than males, suggesting a sex‐based influence on gut microbiota. It has been shown that the sex of the host has a strong effect on the diversity and structure of the microbiome with commonly reported differences associated with different relative abundance of the most represented phyla and genera (Vasconcelos et al. 2023). The present results confirm these observations. Interestingly, the genus *Corynebacterium* was found to be more abundant in females of *P. lusitanicus* , both in this study and in another assessment of the gut microbiota of coastal populations of this species in Northern Portugal (Vasconcelos et al. 2023). This suggests that *Corynebacterium* may serve as a potential biomarker for sex‐specific differences. High abundance of *Corynebacterium* spp. has been correlated with higher digestive efficiency in chickens (Wen et al. 2021), but also with reduced reproductive performance in female birds (Leclaire et al. 2023). This suggests that the abundance of the genus may have implications for reproductive health. The higher abundance of *Corynebacterium* in *P. lusitanicus* females may be influenced by several factors, including hormonal fluctuations, differences in immune system function or behavioural aspects that affect microbial exposure and colonisation. For example, the abundance of *Corynebacterium* in *Calidris* shorebirds varies across geographical regions (Zhang et al. 2021), with this being attributed to differences in physiological stages, such as newly arrived migrants versus breeding individuals, as well as differences in diet composition and environmental factors that can also influence the gut microbiome. In contrast, lower abundances of *Corynebacterium* in males may be linked to higher levels of stress as proposed in mice (He et al. 2024). Mating behaviour may also contribute to the higher diversity in females, with White et al. (2011) reporting that polyandrous female lizards harboured more diverse cloacal bacterial communities than monandrous females, likely through sexual transmission from multiple mates. These results emphasise the need for more research into the functional roles of sex‐associated microbes, such as *Corynebacterium*, in vertebrate reproduction. Overall, these findings underscore the importance of considering host sex as a key factor shaping gut microbial communities. The consistent microbial differences between males and females in several species likely reflect underlying hormonal, immune and behavioural differences that influence microbial colonisation (Org et al. 2015; Markle et al. 2013; Gao et al. 2022; Santos‐Marcos et al. 2023; Le Bras 2024).

In addition to sex‐based effects, we found a positive relationship between body size and the relative abundance of *Parabacteroides*, one of the few significant relationships linked to body size in our study. This genus is normally associated with carbohydrate fermentation and has been linked to energy harvesting efficiency in vertebrates (Feng et al. 2025). The increase in *Parabacteroides* observed with increasing body size may reflect ontogenetic changes in diet, since larger individuals often consume more or different types of prey, which may favour the proliferation of certain microbial taxa. This interpretation aligns with previous research in lizards, where body size has been correlated with microbial diversity or shifts in specific taxa, likely due to changes in diet, metabolism, or habitat use (Kohl et al. 2014; Vasconcelos et al. 2023). Although body size was identified as an influencing factor for only a limited part of the variation in the microbiota in this study, it highlights the potential role of host developmental stage and morphology in shaping microbial communities within wild lizard populations.

## 6 Concluding Remarks

Although both diet (Simões et al. 2025) and gut microbiome (this study) of *P. lusitanicus* are shaped by fire‐induced habitat changes, variation in diet composition was not a strong predictor of gut microbiome structure. Among the factors shaping microbial communities, sex consistently emerged as a significant determinant, with clear differences in composition and diversity. The association between body size and microbial composition, while present, was relatively minor. In contrast, fire regime had a more pronounced effect, particularly on community structure, suggesting that environmental variation plays an important role in shaping the gut microbiome of this species. Interestingly, despite compositional shifts associated with fire history, alpha‐diversity remained relatively stable across fire regimes. This stability in microbial richness, coupled with shifts in community structure, points to a degree of microbial resilience and functional redundancy, which may buffer the host against ecological perturbations. However, it is important to consider that sampling occurred approximately 1 year after the most recent fire events, a temporal gap that may have allowed for partial microbial recovery and could influence interpretations of resilience.

Overall, these patterns align with growing evidence that gut microbial communities are influenced by a combination of external factors (e.g., habitat, diet) and internal intrinsic factors, such as host physiology, age, or sex hormones (Kohl and Yahn 2016; Amato 2016; Xavier et al. 2020). Additionally, the relative independence between diet and microbiome composition observed in *P. lusitanicus* and the lack of significant changes to the functional profile of gut microbiota underscore the adaptability of the microbial communities in the face of ecological perturbations.

Our study provides important insights into the links between fire regimes and gut microbial communities, yet further work is needed to establish causality and uncover the mechanisms driving these patterns. For example, regular longitudinal sampling after fires would better capture microbial responses and trajectory to recovery. It would also be valuable in future studies to include fine‐scale habitat characteristics such as vegetation composition, as well as changes in prey availability over time, to better understand the patterns of variation in microbiome diversity. Integrating microbiome data with host physiological measures (such as stress hormones and immune markers) and functional microbial assays (metagenomics, metatranscriptomics) would also allow for a more comprehensive understanding of how fire shapes host‐microbiome interactions and the consequences for host health and fitness.

These insights emphasise the need to consider both environmental context and host biology when assessing microbiome responses to disturbance, and they provide a foundation for future studies on microbial resilience and host‐microbiome interactions in changing landscapes.

## Author Contributions

**Diana S. Vasconcelos** conceived and designed the research, carried out fieldwork, performed the research, analysed the data, prepared figures and/or tables, authored or reviewed drafts of the article, and approved the final draft. **David James Harris** conceived and designed the research, carried out fieldwork, performed the research, prepared figures and/or tables, authored or reviewed drafts of the article, and approved the final draft. **Catarina Simões** carried out fieldwork, reviewed drafts of the article and approved the final draft. **Catarina Rato** performed the research, prepared figures and/or tables, authored or reviewed drafts of the article and approved the final draft. **Pedro Tarroso** performed the research, prepared figures and/or tables, authored or reviewed drafts of the article and approved the final draft. **Xavier Santos** conceived and designed the research, carried out fieldwork, performed the research, prepared figures and/or tables, authored or reviewed drafts of the article and approved the final draft. **Raquel Xavier** conceived and designed the research, performed the research, analysed data, prepared figures and/or tables, authored or reviewed drafts of the article and approved the final draft.

## Funding

Raquel Xavier was supported by FCT under the Programa Operacional Potencial Humano–Quadro de Referência Estratégico Nacional funds from the European Social Fund and Portuguese Ministério da Educação e Ciência (2020.00854.CEECIND/CP1601/CT0001), Diana S. Vasconcelos was supported by FCT (2022.13485.BD). The work was also supported by FCT project 2022.07460.PTDC (to DJH).

## Disclosure

Field Study Permissions: The following information was supplied relating to field study approvals (i.e., approving body and any reference numbers): Institute for Conservation of Nature and Forests (ICNF), Licence 552–553/2022/CAPT.

DNA Deposition: The following information was supplied regarding the deposition of DNA sequences: The raw sequence reads are available at NCBI's Short Read Archive: PRJNA1391492.

## Ethics Statement

The following information was supplied relating to ethical approvals (i.e., approving body and any reference numbers): Experimental protocols and research were approved by the Portuguese Institute for Conservation of Nature and Forests (ICNF).

## Conflicts of Interest

The authors declare no conflicts of interest.

## Supporting information


**Figure S1:** Comparison of observed versus expected abundances in the mock microbial community after DADA2 processing.
**Figure S2:** Canonical Analysis of Principal Coordinates (CAP) ordinations of gut microbiome composition based on weighted UniFrac and Bray‐Curtis dissimilarities.
**Figure S3:** Gut microbiome composition comparison between male and female *Podarcis lusitanicus* at the phylum level.
**Figure S4:** Relationship between snout‐vent length (SVL) and the relative abundance of Parabacteroides. Each point represents an individual lizard, with colours distinguishing different groups (e.g., sex). The blue regression line indicates a slight positive trend. The shaded area represents the confidence interval of the regression model.
**Table S1:** Variables and corresponding values used to categorise different factors (sex and fire regime) in generalised linear model analyses.
**Table S2:** Most abundant bacterial genera (or highest resolved taxonomic level) across all samples based on mean relative abundance.
**Table S3:** PERMANOVA pairwise comparisons testing the effects of fire history, sex, and body size on gut microbiome beta diversity using three distance metrics (Unweighted UniFrac, Weighted UniFrac, and Bray‐Curtis).

**Data S1:** Supplemental script file with code for the bioinformatics and statistical analyses described in this manuscript.

## Data Availability

The data that support the findings of this study are openly available in NCBIs Short Read Archive under BioProject accession number PRJNA1391492 (will be also available after publication on: https://www.ncbi.nlm.nih.gov/sra/PRJNA1391492). The R scripts used in this microbiome analysis are available as Supporting Information.

## References

[mec70255-bib-0001] Albery, G. F. , I. Turilli , M. B. Joseph , J. Foley , C. H. Frere , and S. Bansal . 2021. “From Flames to Inflammation: How Wildfires Affect Patterns of Wildlife Disease.” Fire Ecology 17: 1–17.

[mec70255-bib-0002] Amato, K. R. 2016. “Incorporating the Gut Microbiota Into Models of Human and Non‐Human Primate Ecology and Evolution.” American Journal of Physical Anthropology 159: 196–215. 10.1002/ajpa.22908 26808106

[mec70255-bib-0003] Amato, K. R. , C. J. Yeoman , A. Kent , et al. 2013. “Habitat Degradation Impacts Black Howler Monkey ( *Alouatta pigra* ) Gastrointestinal Microbiomes.” ISME Journal 7, no. 7: 1344–1353. 23486247 10.1038/ismej.2013.16 PMC3695285

[mec70255-bib-0004] Arakelyan, M. , T. Harutyunyan , S. A. Aghayan , and M. A. Carretero . 2019. “Infection of Parthenogenetic Lizards by Blood Parasites Does Not Support the ‘Red Queen Hypothesis’ but Reveals the Costs of Sex.” Zoology 136: 125709. 31539860 10.1016/j.zool.2019.125709

[mec70255-bib-0005] Arnold, E. N. 1987. “Resource Partition Among Lacertid Lizards in Southern Europe.” Journal of Zoology 1, no. 4: 739–782.

[mec70255-bib-0006] Baldo, L. , J. L. Riera , K. Mitsi , and J. L. Pretus . 2018. “Processes Shaping Gut Microbiota Diversity in Allopatric Populations of the Endemic Lizard *Podarcis lilfordi* From Menorcan Islets (Balearic Islands).” FEMS Microbiology Ecology 94, no. 2: fix186. 10.1093/femsec/fix186 29294010

[mec70255-bib-0007] Benjamini, Y. , and Y. Hochberg . 1995. “Controlling the False Discovery Rate: A Practical and Powerful Approach to Multiple Testing.” Journal of the Royal Statistical Society: Series B (Methodological) 57, no. 1: 289–300.

[mec70255-bib-0008] Beranek, C. T. , A. J. Hamer , S. V. Mahony , et al. 2023. “Severe Wildfires Promoted by Climate Change Negatively Impact Forest Amphibian Metacommunities.” Diversity and Distributions 29, no. 6: 785–800.

[mec70255-bib-0009] Blakeley‐Ruiz, J. A. , A. Bartlett , A. S. McMillan , et al. 2025. “Dietary Protein Source Alters Gut Microbiota Composition and Function.” ISME Journal 19: wraf048. 40116459 10.1093/ismejo/wraf048 PMC12066410

[mec70255-bib-0010] Bletz, M. C. , D. J. Goedbloed , E. Sanchez , et al. 2016. “Amphibian Gut Microbiota Shifts Differentially in Community Structure but Converges on Habitat‐Specific Predicted Functions.” Nature Communications 7, no. 1: 13699. 10.1038/ncomms13699 PMC5171763 27976718

[mec70255-bib-0011] Bolyen, E. , J. R. Rideout , M. R. Dillon , et al. 2019. “Reproducible, Interactive, Scalable and Extensible Microbiome Data Science Using QIIME 2.” Nature Biotechnology 37, no. 8: 852–857. 10.1038/s41587-019-0209-9 PMC7015180 31341288

[mec70255-bib-0012] Bond, W. J. , F. I. Woodward , and G. F. Midgley . 2005. “The Global Distribution of Ecosystems in a World Without Fire.” New Phytologist 165, no. 2: 525–538. 15720663 10.1111/j.1469-8137.2004.01252.x

[mec70255-bib-0013] Bowd, E. J. , E. Egidi , D. B. Lindenmayer , et al. 2022. “Direct and Indirect Effects of Fire on Microbial Communities in a Pyrodiverse Dry‐Sclerophyll Forest.” Journal of Ecology 110, no. 7: 1687–1703.

[mec70255-bib-0014] Bowman, D. M. , C. A. Kolden , J. T. Abatzoglou , F. H. Johnston , G. R. van der Werf , and M. Flannigan . 2020. “Vegetation Fires in the Anthropocene.” Nature Reviews Earth & Environment 1, no. 10: 500–515.

[mec70255-bib-0015] Bray, J. R. , and J. T. Curtis . 1957. “An Ordination of the Upland Forest Communities of Southern Wisconsin.” Ecological Monographs 27, no. 4: 325–349.

[mec70255-bib-0016] Buddle, C. M. , D. W. Langor , G. R. Pohl , and J. R. Spence . 2006. “Arthropod Responses to Harvesting and Wildfire: Implications for Emulation of Natural Disturbance in Forest Management.” Biological Conservation 128, no. 3: 346–357.

[mec70255-bib-0017] Bunker, M. E. , A. E. Arnold , and S. L. Weiss . 2022. “Wild Microbiomes of Striped Plateau Lizards Vary With Reproductive Season, Sex, and Body Size.” Scientific Reports 12, no. 1: 20643. 36450782 10.1038/s41598-022-24518-6 PMC9712514

[mec70255-bib-0018] Bunker, M. E. , M. O. Martin , and S. L. Weiss . 2022. “Recovered Microbiome of an Oviparous Lizard Differs Across Gut and Reproductive Tissues, Cloacal Swabs, and Faeces.” Molecular Ecology Resources 22, no. 5: 1693–1705. 34894079 10.1111/1755-0998.13573

[mec70255-bib-0019] Callahan, B. J. , P. J. McMurdie , and S. P. Holmes . 2017. “Exact Sequence Variants Should Replace Operational Taxonomic Units in Marker‐Gene Data Analysis.” ISME Journal 11, no. 12: 2639–2643. 28731476 10.1038/ismej.2017.119 PMC5702726

[mec70255-bib-0020] Callahan, B. J. , P. J. McMurdie , M. J. Rosen , A. W. Han , A. J. A. Johnson , and S. P. Holmes . 2016. “DADA2: High‐Resolution Sample Inference From Illumina Amplicon Data.” Nature Methods 13, no. 7: 581–583. 27214047 10.1038/nmeth.3869 PMC4927377

[mec70255-bib-0021] Carretero, M. A. , P. Galán , and A. Salvador . 2015. “Lagartija lusitana–*Podarcis guadarramae* (Boscá, 1916).” edited by Enciclopedia Virtual de los Vertebrados Españoles. Museo Nacional de Ciencias Naturales, Madrid.

[mec70255-bib-0022] Carvalho, F. , A. Pradhan , N. Abrantes , et al. 2019. “Wildfire Impacts on Freshwater Detrital Food Webs Depend on Runoff Load, Exposure Time and Burnt Forest Type.” Science of the Total Environment 692: 691–700. 31539977 10.1016/j.scitotenv.2019.07.265

[mec70255-bib-0023] Celi, P. , A. J. Cowieson , F. Fru‐Nji , R. E. Steinert , A. M. Kluenter , and V. Verlhac . 2017. “Gastrointestinal Functionality in Animal Nutrition and Health: New Opportunities for Sustainable Animal Production.” Animal Feed Science and Technology 234: 88–100.

[mec70255-bib-0024] Certini, G. 2005. “Effects of Fire on Properties of Forest Soils: A Review.” Oecologia 143: 1–10. 15688212 10.1007/s00442-004-1788-8

[mec70255-bib-0025] Chiarello, M. , J. C. Auguet , Y. Bettarel , et al. 2018. “Skin Microbiome of Coral Reef Fish Is Highly Variable and Driven by Host Phylogeny and Diet.” Microbiome 6, no. 1: 147. 30143055 10.1186/s40168-018-0530-4 PMC6109317

[mec70255-bib-0026] Colston, T. J. 2017. “Gut Microbiome Transmission in Lizards.” Molecular Ecology 26: 972–974. 28239927 10.1111/mec.13987

[mec70255-bib-0027] Colston, T. J. , and C. R. Jackson . 2016. “Microbiome Evolution Along Divergent Branches of the Vertebrate Tree of Life: What Is Known and Unknown.” Molecular Ecology 25, no. 16: 3776–3800. 27297628 10.1111/mec.13730

[mec70255-bib-0028] Cryan, J. F. , and T. G. Dinan . 2012. “Mind‐Altering Microorganisms: The Impact of the Gut Microbiota on Brain and Behaviour.” Nature Reviews Neuroscience 13, no. 10: 701–712. 22968153 10.1038/nrn3346

[mec70255-bib-0029] Dalias, P. , E. Hadjisterkotis , M. Omirou , et al. 2024. “Wildfire Effects on the Soil Respiration and Bacterial Microbiota Composition in Mediterranean‐Type Ecosystems.” Fire 7, no. 7: 213.

[mec70255-bib-0030] de Jonge, N. , B. Carlsen , M. H. Christensen , C. Pertoldi , and J. L. Nielsen . 2022. “The Gut Microbiome of 54 Mammalian Species.” Frontiers in Microbiology 13: 886252. 35783446 10.3389/fmicb.2022.886252 PMC9246093

[mec70255-bib-0031] Delsuc, F. , J. L. Metcalf , L. Wegener Parfrey , S. J. Song , A. González , and R. Knight . 2014. “Convergence of Gut Microbiomes in Myrmecophagous Mammals.” Molecular Ecology 23, no. 6: 1301–1317. 24118574 10.1111/mec.12501

[mec70255-bib-0032] Deng, Z. , K. Hou , J. Zhao , and H. Wang . 2022. “The Probiotic Properties of Lactic Acid Bacteria and Their Applications in Animal Husbandry.” Current Microbiology 79: 1–11. 10.1007/s00284-021-02722-3 34905106

[mec70255-bib-0033] Diego‐Rasilla, F. J. , and V. Perez‐Mellado . 2003. “Home Range and Habitat Selection by *Podarcis hispanica* (Squamata, Lacertidae) in Western Spain.” Folia ZOOLOGICA‐Praha 52, no. 1: 87–98.

[mec70255-bib-0034] Dong, W. J. , X. Z. Long , X. W. Yang , X. Y. Han , L. Y. Cui , and Q. Tong . 2024. “Impact of Wildfire Ash on Skin and Gut Microbiomes and Survival of *Rana dybowskii* .” Journal of Hazardous Materials 474: 134729. 38805811 10.1016/j.jhazmat.2024.134729

[mec70255-bib-0035] Dooley, S. R. , and K. K. Treseder . 2012. “The Effect of Fire on Microbial Biomass: A Meta‐Analysis of Field Studies.” Biogeochemistry 109: 49–61.

[mec70255-bib-0036] Douglas, G. M. , V. J. Maffei , J. Zaneveld , et al. 2019. “PICRUSt2: An Improved and Extensible Approach for Metagenome Inference.” *bioRxiv* 672295.

[mec70255-bib-0037] Driscoll, D. A. , D. Armenteras , A. F. Bennett , et al. 2021. “How Fire Interacts With Habitat Loss and Fragmentation.” Biological Reviews 96, no. 3: 976–998. 33561321 10.1111/brv.12687

[mec70255-bib-0038] Faith, D. P. 1992. “Conservation Evaluation and Phylogenetic Diversity.” Biological Conservation 61, no. 1: 1–10.

[mec70255-bib-0039] Fan, C. , L. Zhang , S. Jia , et al. 2022. “Seasonal Variations in the Composition and Functional Profiles of Gut Microbiota Reflect Dietary Changes in Plateau Pikas.” Integrative Zoology 17, no. 3: 379–395. 35051309 10.1111/1749-4877.12630 PMC9305894

[mec70255-bib-0040] Feng, X. , Y. Liu , S. Xu , et al. 2025. “Functional Analysis of *Parabacteroides distasonis* F4: A Novel Probiotic Strain Linked to Calf Growth and Rumen Fermentation.” Journal of Animal Science and Biotechnology 16, no. 1: 50. 40181465 10.1186/s40104-025-01182-0 PMC11969818

[mec70255-bib-0041] Ferreira, D. , C. Mateus , and X. Santos . 2016. “Responses of Reptiles to Fire in Transition Zones Are Mediated by Bioregion Affinity of Species.” Biodiversity and Conservation 25: 1543–1557.

[mec70255-bib-0042] Ferreira, D. , C. Pinho , J. C. Brito , and X. Santos . 2019. “Increase of Genetic Diversity Indicates Ecological Opportunities in Recurrent‐Fire Landscapes for Wall Lizards.” Scientific Reports 9, no. 1: 5383. 30926838 10.1038/s41598-019-41729-6 PMC6441018

[mec70255-bib-0043] Ferrenberg, S. , S. P. O'Neill , J. E. Knelman , et al. 2013. “Changes in Assembly Processes in Soil Bacterial Communities Following a Wildfire Disturbance.” ISME Journal 7, no. 6: 1102–1111. 23407312 10.1038/ismej.2013.11 PMC3660671

[mec70255-bib-0044] Fields, R. D. 2008. “White Matter in Learning, Cognition and Psychiatric Disorders.” Trends in Neurosciences 31, no. 7: 361–370. 18538868 10.1016/j.tins.2008.04.001 PMC2486416

[mec70255-bib-0045] Fromm, E. , L. Zinger , F. Pellerin , et al. 2024. “Warming Effects on Lizard Gut Microbiome Depend on Habitat Connectivity.” Proceedings of the Royal Society B 291, no. 2021: 20240220. 38654642 10.1098/rspb.2024.0220 PMC11040258

[mec70255-bib-0046] Gao, Y. , P. Wu , S. Cui , A. Ali , and G. Zheng . 2022. “Divergence in Gut Bacterial Community Between Females and Males in the Wolf Spider *Pardosa astrigera* .” Ecology and Evolution 12, no. 4: e8823. 35432934 10.1002/ece3.8823 PMC9005928

[mec70255-bib-0047] Gomes, F. , G. E. Furtado , M. Henriques , et al. 2022. “The Skin Microbiome of Infected Pressure Ulcers: A Review and Implications for Health Professionals.” European Journal of Clinical Investigation 52, no. 1: e13688. 34601718 10.1111/eci.13688

[mec70255-bib-0048] Gongalsky, K. B. , and T. Persson . 2013. “Recovery of Soil Macrofauna After Wildfires in Boreal Forests.” Soil Biology and Biochemistry 57: 182–191.

[mec70255-bib-0049] Hammer, T. J. , and M. D. Bowers . 2015. “Gut Microbes May Facilitate Insect Herbivory of Chemically Defended Plants.” Oecologia 179, no. 1: 1–14. 25936531 10.1007/s00442-015-3327-1

[mec70255-bib-0050] He, H. , Z. Zhao , C. Xiao , et al. 2024. “Gut Microbiome Promotes Mice Recovery From Stress‐Induced Depression by Rescuing Hippocampal Neurogenesis.” Neurobiology of Disease 191: 106396. 38176570 10.1016/j.nbd.2023.106396

[mec70255-bib-0051] Hernández, M. , S. Ancona , S. Hereira‐Pacheco , A. H. Díaz de la Vega‐Pérez , A. Alberdi , and Y. E. Navarro‐Noya . 2024. “Seasonal Dietary Changes Relate to Gut Microbiota Composition Depending on the Host Species but Do Not Correlate With Gut Microbiota Diversity in Arthropod‐Eating Lizards.” Molecular Ecology 33: e17426. 38825980 10.1111/mec.17426

[mec70255-bib-0052] Hetem, R. S. , A. Fuller , S. K. Maloney , and D. Mitchell . 2014. “Responses of Large Mammals to Climate Change.” Temperature 1, no. 2: 115–127. 10.4161/temp.29651 PMC4977165 27583293

[mec70255-bib-0053] Hoffbeck, C. , D. M. Middleton , S. K. Lamar , S. N. Keall , N. J. Nelson , and M. W. Taylor . 2024. “Gut Microbiome of the Sole Surviving Member of Reptile Order *Rhynchocephalia* Reveals Biogeographic Variation, Influence of Host Body Condition and a Substantial Core Microbiota in Tuatara Across New Zealand.” Ecology and Evolution 14, no. 2: e11073. 38405409 10.1002/ece3.11073 PMC10884523

[mec70255-bib-0054] Hong, P. Y. , E. Wheeler , I. K. Cann , and R. I. Mackie . 2011. “Phylogenetic Analysis of the Fecal Microbial Community in Herbivorous Land and Marine Iguanas of the Galápagos Islands Using 16S rRNA‐Based Pyrosequencing.” ISME Journal 5, no. 9: 1461–1470. 21451584 10.1038/ismej.2011.33 PMC3160690

[mec70255-bib-0055] Hossack, B. R. , and D. S. Pilliod . 2011. “Amphibian Responses to Wildfire in the Western United States: Emerging Patterns From Short‐Term Studies.” Fire Ecology 7, no. 2: 129–144.

[mec70255-bib-0056] Jolly, C. J. , C. R. Dickman , T. S. Doherty , et al. 2022. “Animal Mortality During Fire.” Global Change Biology 28, no. 6: 2053–2065. 34989061 10.1111/gcb.16044

[mec70255-bib-0057] Kanehisa, M. , Y. Sato , M. Furumichi , and K. Morishima . 2019. “Tanabe, M New Approach for Understanding Genome Variations in KEGG.” Nucleic Acids Research 47: D590–D595. 30321428 10.1093/nar/gky962 PMC6324070

[mec70255-bib-0058] Kelly, L. T. , K. M. Giljohann , A. Duane , et al. 2020. “Fire and Biodiversity in the Anthropocene.” Science 370, no. 6519: eabb0355. 33214246 10.1126/science.abb0355

[mec70255-bib-0059] Kembel, S. W. , P. D. Cowan , M. R. Helmus , et al. 2010. “Picante: R Tools for Integrating Phylogenies and Ecology.” Bioinformatics 26, no. 11: 1463–1464. 20395285 10.1093/bioinformatics/btq166

[mec70255-bib-0060] Kohl, K. D. , R. B. Weiss , J. Cox , C. Dale , and M. Denise Dearing . 2014. “Gut Microbes of Mammalian Herbivores Facilitate Intake of Plant Toxins.” Ecology Letters 17, no. 10: 1238–1246. 25040855 10.1111/ele.12329

[mec70255-bib-0061] Kohl, K. D. , and J. Yahn . 2016. “Effects of Environmental Temperature on the Gut Microbial Communities of Tadpoles.” Environmental Microbiology 18, no. 5: 1561–1565. 26940397 10.1111/1462-2920.13255

[mec70255-bib-0062] Kozich, J. J. , S. L. Westcott , N. T. Baxter , S. K. Highlander , and P. D. Schloss . 2013. “Development of a Dual‐Index Sequencing Strategy and Curation Pipeline for Analyzing Amplicon Sequence Data on the MiSeq Illumina Sequencing Platform.” Applied and Environmental Microbiology 79, no. 17: 5112–5120. 23793624 10.1128/AEM.01043-13 PMC3753973

[mec70255-bib-0063] Lazarkevich, I. , S. Engibarov , S. Mitova , et al. 2024. “16S rRNA Gene Sequencing‐Based Identification and Comparative Analysis of the Fecal Microbiota of Five Syntopic Lizard Species From a Low‐Mountain Area in Western Bulgaria.” Applied Microbiology 4, no. 1: 181–193.

[mec70255-bib-0064] Le Bras, A. 2024. “Sex Differences in Microbiota.” Lab Animal 53, no. 9: 218.

[mec70255-bib-0065] Leclaire, S. , M. Pineaux , P. Blanchard , J. White , and S. A. Hatch . 2023. “Microbiota Composition and Diversity of Multiple Body Sites Vary According to Reproductive Performance in a Seabird.” Molecular Ecology 32, no. 9: 2115–2133. 35152516 10.1111/mec.16398

[mec70255-bib-0066] Legendre, P. L. , and L. L. Legendre . 2012. Numerical Ecology. 3rd ed. Elsevier.

[mec70255-bib-0067] Lemieux‐Labonté, V. , C. Vigliotti , Z. Tadic , et al. 2022. “Proximate Drivers of Population‐Level Lizard Gut Microbial Diversity: Impacts of Diet, Insularity, and Local Environment.” Microorganisms 10, no. 8: 1550. 36013968 10.3390/microorganisms10081550 PMC9413874

[mec70255-bib-0068] Li, L. , Y. Su , F. Li , et al. 2020. “The Effects of Daily Fasting Hours on Shaping Gut Microbiota in Mice.” BMC Microbiology 20: 1–8. 32209070 10.1186/s12866-020-01754-2 PMC7092480

[mec70255-bib-0069] Li, Y. , Y. Yan , H. Fu , et al. 2023. “Does Diet or Macronutrients Intake Drive the Structure and Function of Gut Microbiota?” Frontiers in Microbiology 14: 1126189. 36860485 10.3389/fmicb.2023.1126189 PMC9970161

[mec70255-bib-0071] Love, M. I. , W. Huber , and S. Anders . 2014. “Moderated Estimation of Fold Change and Dispersion for RNA‐Seq Data With DESeq2.” Genome Biology 15, no. 12: 550. 25516281 10.1186/s13059-014-0550-8 PMC4302049

[mec70255-bib-0072] Lozupone, C. , and R. Knight . 2005. “UniFrac: A New Phylogenetic Method for Comparing Microbial Communities.” Applied and Environmental Microbiology 71, no. 12: 8228–8235. 16332807 10.1128/AEM.71.12.8228-8235.2005 PMC1317376

[mec70255-bib-0073] Lozupone, C. A. , M. Hamady , S. T. Kelley , and R. Knight . 2007. “Quantitative and Qualitative Beta Diversity Measures Lead to Different Insights Into Factors That Structure Microbial Communities.” Applied and Environmental Microbiology 73, no. 5: 1576–1585. 17220268 10.1128/AEM.01996-06 PMC1828774

[mec70255-bib-0074] Mansoor, S. , I. Farooq , M. M. Kachroo , et al. 2022. “Elevation in Wildfire Frequencies With Respect to the Climate Change.” Journal of Environmental Management 301: 113769. 34600426 10.1016/j.jenvman.2021.113769

[mec70255-bib-0075] Markle, J. G. , D. N. Frank , S. Mortin‐Toth , et al. 2013. “Sex Differences in the Gut Microbiome Drive Hormone‐Dependent Regulation of Autoimmunity.” Science 339, no. 6123: 1084–1088. 23328391 10.1126/science.1233521

[mec70255-bib-0076] Marques Silva, S. , R. Xavier , A. C. R. Gomes , P. Beltrão , G. C. Cardoso , and S. Trigo . 2024. “Phenotypic Associations of Common Waxbill Gut and Feather Microbiome Diversity in a Shared Environment.” Biological Journal of the Linnean Society 141, no. 2: 184–190.

[mec70255-bib-0077] Martin, B. D. , D. Witten , and A. D. Willis . 2020. “Modeling Microbial Abundances and Dysbiosis With Beta‐Binomial Regression.” Annals of Applied Statistics 14, no. 1: 94. 32983313 10.1214/19-aoas1283 PMC7514055

[mec70255-bib-0078] McMurdie, P. J. , and S. Holmes . 2013. “Phyloseq: An R Package for Reproducible Interactive Analysis and Graphics of Microbiome Census Data.” PLoS One 8, no. 4: e61217. 23630581 10.1371/journal.pone.0061217 PMC3632530

[mec70255-bib-0079] McMurdie, P. J. , and S. Holmes . 2014. “Waste Not, Want Not: Why Rarefying Microbiome Data Is Inadmissible.” PLoS Computational Biology 10, no. 4: e1003531. 24699258 10.1371/journal.pcbi.1003531 PMC3974642

[mec70255-bib-0080] Montoya‐Ciriaco, N. , S. Gómez‐Acata , L. C. Muñoz‐Arenas , et al. 2020. “Dietary Effects on Gut Microbiota of the Mesquite Lizard *Sceloporus grammicus* (Wiegmann, 1828) Across Different Altitudes.” Microbiome 8: 1–19. 31980039 10.1186/s40168-020-0783-6 PMC6982387

[mec70255-bib-0081] Moretti, M. , M. K. Obrist , and P. Duelli . 2004. “Arthropod Biodiversity After Forest Fires: Winners and Losers in the Winter Fire Regime of the Southern Alps.” Ecography 27, no. 2: 173–186.

[mec70255-bib-0082] Nelson, A. R. , A. B. Narrowe , C. C. Rhoades , et al. 2022. “Wildfire‐Dependent Changes in Soil Microbiome Diversity and Function.” Nature Microbiology 7, no. 9: 1419–1430. 10.1038/s41564-022-01203-y PMC9418001 36008619

[mec70255-bib-0083] O'Keeffe, K. R. , F. W. Halliday , C. D. Jones , I. Carbone , and C. E. Mitchell . 2021. “Parasites, Niche Modification and the Host Microbiome: A Field Survey of Multiple Parasites.” Molecular Ecology 30, no. 10: 2404–2416. 33740826 10.1111/mec.15892

[mec70255-bib-0084] Oksanen, J. , F. G. Blanchet , R. Kindt , et al. 2013. “Package ‘vegan’.” Community Ecology Package, version, 2(9), 1–295.

[mec70255-bib-0085] Olsson, M. , E. Wapstra , T. Madsen , and B. Silverin . 2000. “Testosterone, Ticks and Travels: A Test of the Immunocompetence‐Handicap Hypothesis in Free‐Ranging Male Sand Lizards.” Proceedings of the Royal Society of London. Series B: Biological Sciences 267, no. 1459: 2339–2343. 10.1098/rspb.2000.1289 PMC1690810 11413653

[mec70255-bib-0086] Org, E. , M. Mehrabian , and A. J. Lusis . 2015. “Unraveling the Environmental and Genetic Interactions in Atherosclerosis: Central Role of the Gut Microbiota.” Atherosclerosis 241, no. 2: 387–399. 26071662 10.1016/j.atherosclerosis.2015.05.035 PMC4510029

[mec70255-bib-0087] Pereira, A. , M. C. Soares , T. Santos , et al. 2023. “Reef Location and Client Diversity Influence the Skin Microbiome of the Caribbean Cleaner Goby *Elacatinus evelynae* .” Microbial Ecology 85, no. 2: 372–382. 35275230 10.1007/s00248-022-01984-z

[mec70255-bib-0088] Pinheiro, J. , D. Bates , S. DebRoy , D. Sarkar , and R Core Team . 2021. “nlme: Linear and Nonlinear Mixed Effects Models.” R Package Version 3.1‐152. https://CRAN.R‐project.org/package=nlme.

[mec70255-bib-0089] Pressler, Y. , J. C. Moore , and M. F. Cotrufo . 2019. “Belowground Community Responses to Fire: Meta‐Analysis Reveals Contrasting Responses of Soil Microorganisms and Mesofauna.” Oikos 128, no. 3: 309–327.

[mec70255-bib-0090] Pruesse, E. , C. Quast , K. Knittel , et al. 2007. “SILVA: A Comprehensive Online Resource for Quality Checked and Aligned Ribosomal RNA Sequence Data Compatible With ARB.” Nucleic Acids Research 35, no. 21: 7188–7196. 17947321 10.1093/nar/gkm864 PMC2175337

[mec70255-bib-0091] Quast, C. , E. Pruesse , P. Yilmaz , et al. 2012. “The SILVA Ribosomal RNA Gene Database Project: Improved Data Processing and Web‐Based Tools.” Nucleic Acids Research 41, no. D1: D590–D596. 23193283 10.1093/nar/gks1219 PMC3531112

[mec70255-bib-0092] R Core Team . 2024. “_R: A Language and Environment for Statistical Computing_.” R Foundation for Statistical Computing, Vienna, Austria. https://www.R‐project.org/.

[mec70255-bib-0093] Rato, C. , L. B. Sreelatha , F. Gómez‐Ramírez , and M. A. Carretero . 2025. “A Pleistocene Biogeography in Miniature: The Small‐Scale Evolutionary History of *Podarcis lusitanicus* (Squamata, Lacertidae).” Journal of Biogeography 52, no. 1: 186–198.

[mec70255-bib-0094] Robinson, N. M. , S. W. Leonard , E. G. Ritchie , et al. 2013. “Refuges for Fauna in Fire‐Prone Landscapes: Their Ecological Function and Importance.” Journal of Applied Ecology 50, no. 6: 1321–1329.

[mec70255-bib-0095] Rosado, D. , P. Canada , S. Marques Silva , N. Ribeiro , P. Diniz , and R. Xavier . 2023. “Disruption of the Skin, Gill, and Gut Mucosae Microbiome of Gilthead Seabream Fingerlings After Bacterial Infection and Antibiotic Treatment.” FEMS Microbes 4: xtad011. 37389204 10.1093/femsmc/xtad011 PMC10306326

[mec70255-bib-0096] Rosado, D. , M. Pérez‐Losada , R. Severino , et al. 2022. “Monitoring Infection and Antibiotic Treatment in the Skin Microbiota of Farmed European Seabass ( *Dicentrarchus labrax* ) Fingerlings.” Microbial Ecology 83: 789–797. 34245329 10.1007/s00248-021-01795-8

[mec70255-bib-0097] Rowland, I. , G. Gibson , A. Heinken , et al. 2018. “Gut Microbiota Functions: Metabolism of Nutrients and Other Food Components.” European Journal of Nutrition 57, no. 1: 1–24. 10.1007/s00394-017-1445-8 PMC5847071 28393285

[mec70255-bib-0098] Sagonas, K. , P. Pafilis , P. Lymberakis , C. M. Donihue , A. Herrel , and E. D. Valakos . 2014. “Insularity Affects Head Morphology, Bite Force and Diet in a Mediterranean Lizard.” Biological Journal of the Linnean Society 112, no. 3: 469–484.

[mec70255-bib-0099] Santos, J. L. , H. Sitters , D. A. Keith , W. L. Geary , R. Tingley , and L. T. Kelly . 2022. “A Demographic Framework for Understanding Fire‐Driven Reptile Declines in the ‘Land of the Lizards’.” Global Ecology and Biogeography 31, no. 10: 2105–2119.

[mec70255-bib-0100] Santos, X. , A. Badiane , and C. Matos . 2016. “Contrasts in Short‐ and Long‐Term Responses of Mediterranean Reptile Species to Fire and Habitat Structure.” Oecologia 180, no. 1: 205–216. 26408003 10.1007/s00442-015-3453-9

[mec70255-bib-0101] Santos‐Marcos, J. A. , M. Mora‐Ortiz , M. Tena‐Sempere , J. Lopez‐Miranda , and A. Camargo . 2023. “Interaction Between Gut Microbiota and Sex Hormones and Their Relation to Sexual Dimorphism in Metabolic Diseases.” Biology of Sex Differences 14, no. 1: 4. 36750874 10.1186/s13293-023-00490-2 PMC9903633

[mec70255-bib-0102] Shapira, M. 2016. “Gut Microbiotas and Host Evolution: Scaling Up Symbiosis.” Trends in Ecology & Evolution 31, no. 7: 539–549. 27039196 10.1016/j.tree.2016.03.006

[mec70255-bib-0103] Simões, C. , D. S. Vasconcelos , R. Xavier , X. Santos , C. Rato , and D. J. Harris . 2025. “From Ashes to Adaptation: The Impact of Wildfires on the Diet of *Podarcis lusitanicus* Revealed by DNA Metabarcoding.” PLoS One 20, no. 10: e0319238. 41032509 10.1371/journal.pone.0319238 PMC12488016

[mec70255-bib-0104] Smith, A. , B. Meulders , C. M. Bull , and D. Driscoll . 2012. “Wildfire‐Induced Mortality of Australian Reptiles.” Herpetology Notes 5: 233–235.

[mec70255-bib-0105] Smith, S. N. , T. J. Colston , and C. D. Siler . 2021. “Venomous Snakes Reveal Ecological and Phylogenetic Factors Influencing Variation in Gut and Oral Microbiomes.” Frontiers in Microbiology 12: 657754. 33841384 10.3389/fmicb.2021.657754 PMC8032887

[mec70255-bib-0106] Song, S. J. , J. G. Sanders , F. Delsuc , et al. 2020. “Comparative Analyses of Vertebrate Gut Microbiomes Reveal Convergence Between Birds and Bats.” MBio 11, no. 1: 10–1128. 10.1128/mBio.02901-19 PMC6946802 31911491

[mec70255-bib-0107] Soria, R. , A. Tortosa , N. Rodríguez‐Berbel , M. E. Lucas‐Borja , R. Ortega , and I. Miralles . 2023. “Short‐Term Response of Soil Bacterial Communities After Prescribed Fires in Semi‐Arid Mediterranean Forests.” Fire 6, no. 4: 145.

[mec70255-bib-0108] Stephens, S. L. , N. Burrows , A. Buyantuyev , et al. 2014. “Temperate and Boreal Forest Mega‐Fires: Characteristics and Challenges.” Frontiers in Ecology and the Environment 12, no. 2: 115–122.

[mec70255-bib-0109] Swengel, A. B. 2001. “A Literature Review of Insect Responses to Fire, Compared to Other Conservation Managements of Open Habitat.” Biodiversity and Conservation 10, no. 7: 1141–1169.

[mec70255-bib-0110] Thaiss, C. A. , N. Zmora , M. Levy , and E. Elinav . 2016. “The Microbiome and Innate Immunity.” Nature 535, no. 7610: 65–74. 27383981 10.1038/nature18847

[mec70255-bib-0111] Tong, Q. , X. N. Liu , Z. F. Hu , et al. 2019. “Effects of Captivity and Season on the Gut Microbiota of the Brown Frog ( *Rana dybowskii* ).” Frontiers in Microbiology 10: 1912. 31507549 10.3389/fmicb.2019.01912 PMC6716059

[mec70255-bib-0112] Tonini, M. , J. Parente , and M. G. Pereira . 2018. “Global Assessment of Rural–Urban Interface in Portugal Related to Land Cover Changes.” Natural Hazards and Earth System Sciences 18, no. 6: 1647–1664.

[mec70255-bib-0113] Underwood, E. C. , and J. F. Quinn . 2010. “Response of Ants and Spiders to Prescribed Fire in Oak Woodlands of California.” Journal of Insect Conservation 14, no. 4: 359–366.

[mec70255-bib-0114] Vasconcelos, D. S. , D. J. Harris , I. Damas‐Moreira , A. Pereira , and R. Xavier . 2023. “Factors Shaping the Gut Microbiome of Five Species of Lizards From Different Habitats.” PeerJ 11: e15146. 37187519 10.7717/peerj.15146 PMC10178224

[mec70255-bib-0115] Wang, Z. , R. Wu , and Y. Yang . 2024. “A Comparison of Digestive Strategies for *Teratoscincus roborowskii* With Different Diet Compositions: Digestive Enzyme Activities, Gut Microbiota, and Metabolites.” Ecology and Evolution 14, no. 12: e70751. 39717646 10.1002/ece3.70751 PMC11663733

[mec70255-bib-0116] Wen, C. , W. Yan , C. Mai , et al. 2021. “Joint Contributions of the Gut Microbiota and Host Genetics to Feed Efficiency in Chickens.” Microbiome 9: 1–23. 34074340 10.1186/s40168-021-01040-x PMC8171024

[mec70255-bib-0125] White, J. , M. Richard , M. Massot , and S. Meylan . 2011. “Cloacal Bacterial Diversity Increases With Multiple Mates: Evidence of Sexual Transmission in Female Common Lizards.” PLoS One 6, no. 7: e22339. 21811590 10.1371/journal.pone.0022339 PMC3141023

[mec70255-bib-0117] Wickham, H. 2016. ggplot2: Elegant Graphics for Data Analysis. Springer‐Verlag. https://ggplot2.tidyverse.org.

[mec70255-bib-0118] Xavier, R. , A. Pereira , A. Pagan , et al. 2020. “The Effects of Environment and Ontogeny on the Skin Microbiome of Two *Stegastes* Damselfishes (Pomacentridae) From the Eastern Caribbean Sea.” Marine Biology 167: 1–12.

[mec70255-bib-0119] Xavier, R. , R. Severino , and S. M. Silva . 2024. “Signatures of Dysbiosis in Fish Microbiomes in the Context of Aquaculture.” Reviews in Aquaculture 16, no. 2: 706–731.

[mec70255-bib-0120] Yang, C. , J. Mai , X. Cao , A. Burberry , F. Cominelli , and L. Zhang . 2023. “ggpicrust2: An R Package for PICRUSt2 Predicted Functional Profile Analysis and Visualization.” Bioinformatics 39, no. 8: btad470. 37527009 10.1093/bioinformatics/btad470 PMC10425198

[mec70255-bib-0121] Youngblut, N. D. , G. H. Reischer , W. Walters , et al. 2019. “Host Diet and Evolutionary History Explain Different Aspects of Gut Microbiome Diversity Among Vertebrate Clades.” Nature Communications 10, no. 1: 2200. 10.1038/s41467-019-10191-3 PMC6522487 31097702

[mec70255-bib-0122] Zhang, Z. , Z. Yang , and L. Zhu . 2021. “Gut Microbiome of Migratory Shorebirds: Current Status and Future Perspectives.” Ecology and Evolution 11, no. 9: 3737–3745. 33976772 10.1002/ece3.7390 PMC8093701

[mec70255-bib-0123] Zhou, J. , T. M. Nelson , C. Rodriguez Lopez , R. R. Sarma , S. J. Zhou , and L. A. Rollins . 2020. “A Comparison of Nonlethal Sampling Methods for Amphibian Gut Microbiome Analyses.” Molecular Ecology Resources 20, no. 4: 844–855. 31990452 10.1111/1755-0998.13139

[mec70255-bib-0124] Zhu, G. , H. Song , M. Duan , et al. 2025. “Dietary Preferences Affect the Gut Microbiota of Three Snake Species (Squamata: Colubridae).” Frontiers in Microbiology 16: 1559646. 40469724 10.3389/fmicb.2025.1559646 PMC12136495

