# Supplementary material for: Shaped by Fire: Unravelling the Impact of Fire on Lizard Gut Microbiome
Source: Mol Ecol. 2026 Jan 28;35(2):e70255. doi: 10.1111/mec.70255 (PMC12853078; doi:10.1111/mec.70255)

Shaped by Fire: Unraveling the Impact of Fire on Lizard Gut Microbiome

**Diana S. Vasconcelos^1,2,3*^, David James Harris^1,3^, Pedro Tarroso^1,3^ , Catarina Simões^1,3^, Catarina Rato ^4^, Xavier Santos^1,3^, Raquel Xavier^1,3*^**

^1^ CIBIO, Centro de Investigação em Biodiversidade e Recursos Genéticos, *InBIO* Laboratório Associado, Campus de Vairão, Universidade do Porto, 4485-661 Vairão, Portugal

^2^ Departamento de Biologia, Faculdade de Ciências, Universidade do Porto, 4099-002 Porto, Portugal

^3^ BIOPOLIS Program in Genomics, Biodiversity and Land Planning, CIBIO, Campus de Vairão, 4485-661 Vairão, Portugal

^4^ APH, Associação Portuguesa de Herpetologia, Porto, Portugal.

*corresponding authors

**SUPPLEMENTARY MATERIAL**
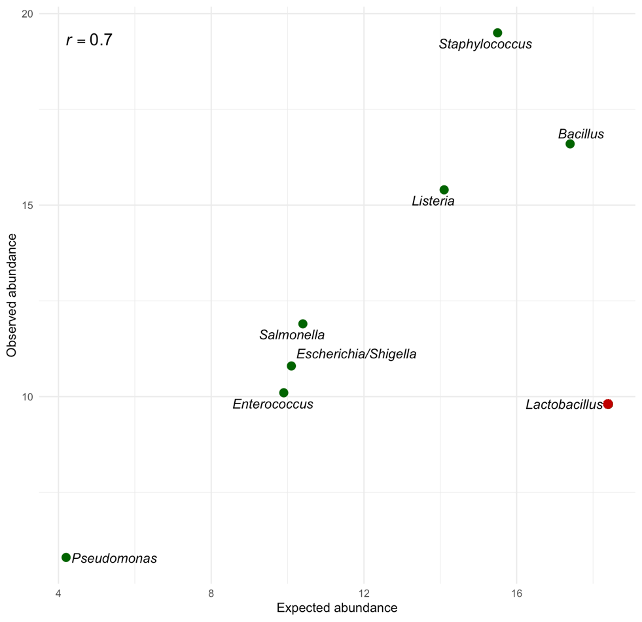


**Figure S1 -** Comparison of observed *versus* expected abundances in the mock microbial community after DADA2 processing. Points represent individual taxa in the mock. Red color indicates deviation from expected values (green = close match, red = outlier). Pearson correlation (r = 0.7) indicates agreement between observed and expected abundances, demonstrating accurate recovery of community composition. Excluding *Lactobacillus*, Pearson correlation increased (r=0.95) showing very high association between observed and expected abundances.

**Table S1**- Variables and corresponding values used to categorise the different factors on GLM (Generalised Linear Model) analysis.

|  | **Variables** | **Value** |
| --- | --- | --- |
| **Sex** | Male vs Male; Female vs Female | 0 |
|  | Male vs Female; Female vs Male | 1 |
| **Fire regime** | B16 vs B16; B22 vs B22; UN vs UN | 0 |
|  | B16 vs B22; B22 vs B16 | 1 |
|  | B16 vs UN; UN vs B16 | 2 |
|  | B22 vs UN; UN vs B22 | 3 |

**Table S2** - Twenty most abundant bacterial genera (or highest resolved taxonomic level) across all samples based on mean relative abundance. Values represent the average percentage of sequences assigned to each genus.

|  | **Taxa** | **MeanRelativeAbundance (%)** |
| --- | --- | --- |
| **Phyla** | Firmicutes | 45,25 |
|  | Bacteroidota | 24,40 |
|  | Actinobacteriota | 12,78 |
|  | Proteobacteria | 9,17 |
|  | Campilobacterota | 3,34 |
| **Genera** | Unclassified Firmicutes | 17.5 |
|  | *Bacteroides* | 16.4 |
|  | Unclassified Actinobacteriota | 11.1 |
|  | *Parabacteroides* | 5.35 |
|  | *Roseburia* | 3.87 |
|  | *Citrobacter* | 3.61 |
|  | *Helicobacter* | 3.34 |
|  | *Anaerosporobacter* | 2.95 |
|  | *Lachnoclostridium* | 2.93 |
|  | Lachnospiraceae_NK4A136_group | 2.67 |
|  | *Coprobacillus* | 1.70 |
|  | Unclassified Proteobacteria | 1.49 |
|  | *Odoribacter* | 1.41 |
|  | Unclassified Cyanobacteria | 1.29 |
|  | *Faecalitalea* | 1.08 |
|  | *Mycoplasma* | 1.08 |
|  | *Hungatella* | 1.07 |
|  | *Lactococcus* | 1.04 |
|  | *Klebsiella* | 0.96 |
|  | *Dielma* | 0.92 |

**Table S3** - Summary of PERMANOVA (adonis2) results testing the effects of fire history, sex, and body size (SVL) on lizard gut microbiome beta diversity. Results are shown for three distance metrics: Unweighted UniFrac, Weighted UniFrac, and Bray-Curtis. Pairwise comparisons for fire history include fire_2016, fire_2022, and unburned sites. Significance is assessed with 9,999 permutations stratified by locality, and p-values are FDR-adjusted. Statistically significant comparisons (adjusted p < 0.05) are highlighted.

|  | **Comparison** | **F_value** | **R2** | **p_value** | **p_value_adj** |
| --- | --- | --- | --- | --- | --- |
| Unweighted UniFrac | **fire_2016_vs_fire_2022** | **1.9564** | **0.0124** | **0.0013** | **0.0039** |
|  | **fire_2016_vs_unburned** | **1.5484** | **0.0105** | **0.0166** | **0.0166** |
|  | **fire_2022_vs_unburned** | **1.7737** | **0.0117** | **0.0044** | **0.0066** |
| Weighted UniFrac | fire_2016_vs_fire_2022 | 2.3384 | 0.0148 | 0.0601 | 0.1224 |
|  | fire_2016_vs_unburned | 1.1487 | 0.0078 | 0.312 | 0.312 |
|  | fire_2022_vs_unburned | 2.0224 | 0.0133 | 0.0816 | 0.1224 |
| Bray-Curtis | **fire_2016_vs_fire_2022** | **2.1752** | **0.0138** | **0.0038** | **0.0114** |
|  | **fire_2016_vs_unburned** | **1.6396** | **0.0111** | **0.045** | **0.045** |
|  | **fire_2022_vs_unburned** | **2.1191** | **0.0139** | **0.0084** | **0.0126** |
| Unweighted UniFrac ~ Sex | **Male_vs_Female** | **1.4592** | **0.00639** | **0.015** | **0.015** |
| Weighted UniFrac ~ Sex | **Male_vs_Female** | **3.0061** | **0.01307** | **0.025** | **0.025** |
| Bray-Curtis ~ Sex | **Male_vs_Female** | **2.3135** | **0.01009** | **0.02** | **0.02** |
| Unweighted UniFrac ~ SVL | **SVL** | \| **2.54** \| \| --- \| | **0.01107** | **0.005** | **0.01** |
| Weighted UniFrac ~ SVL | **SVL** | 3.01 | 0.01309 | 0.050 | 0.05 |
| Bray-Curtis ~ SVL | **SVL** | **2.48** | **0.01081** | **0.005** | **0.01** |

**Figure S2 -** Canonical Analysis of Principal Coordinates (CAP) ordinations of gut microbiome composition based on weighted UniFrac and Bray–Curtis dissimilarities. Points represent individual samples, colored by fire history (Type_fire) and shaped by sex. Ellipses indicate 95% confidence intervals for each fire history group. Titles show the proportion of variance (R²) explained by the constrained model (Type_fire, Sex, SVL).

*
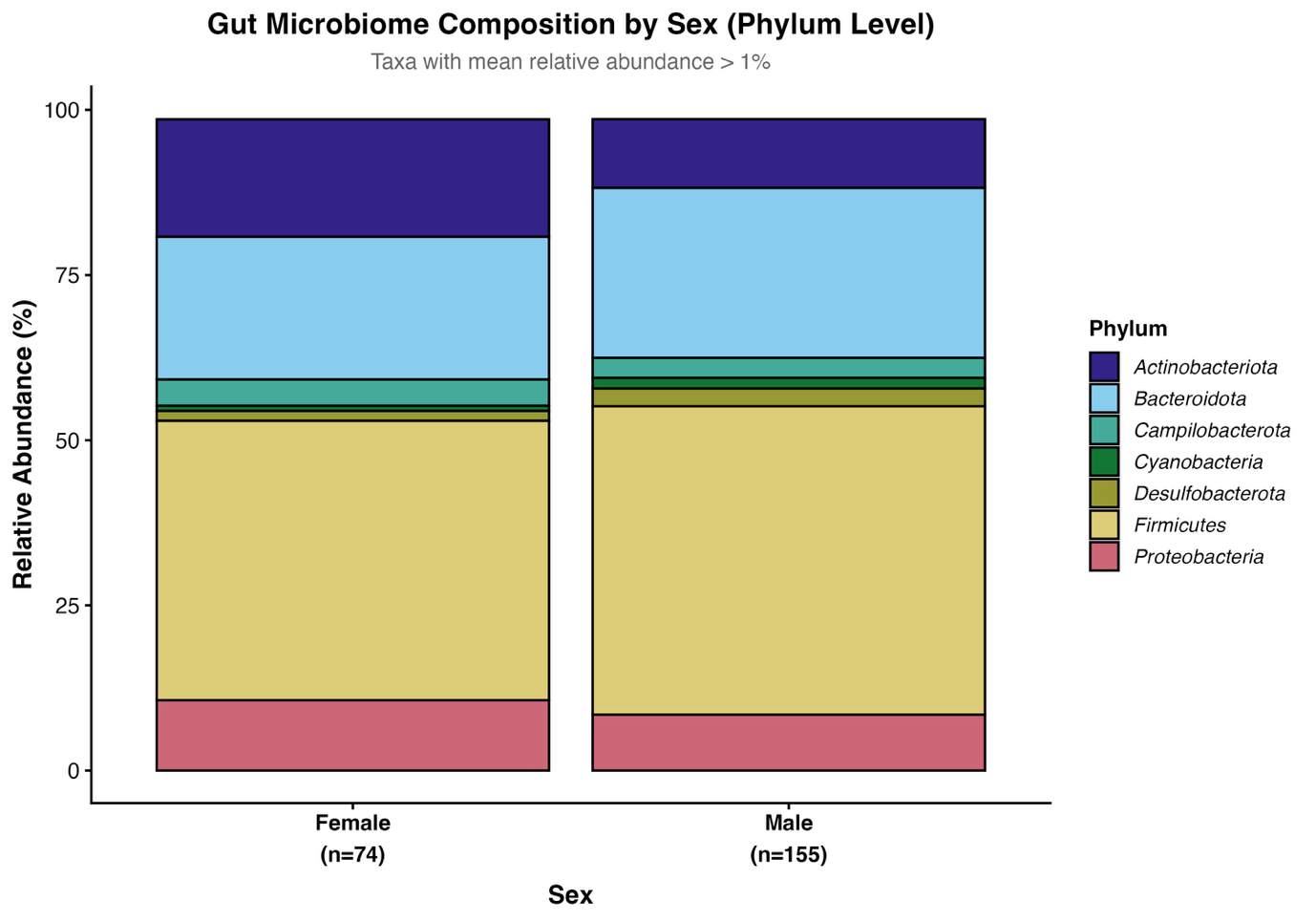
*

**Figure S3 -** Gut microbiome composition comparison between male and female *Podarcis lusitanicus* at the phylum level.

**Figure S4** - Relationship between snout-vent length (SVL) and the relative abundance of Parabacteroides. Each point represents an individual lizard, with colors distinguishing different groups (e.g., sex). The blue regression line indicates a slight positive trend. The shaded area represents the confidence interval of the regression model.


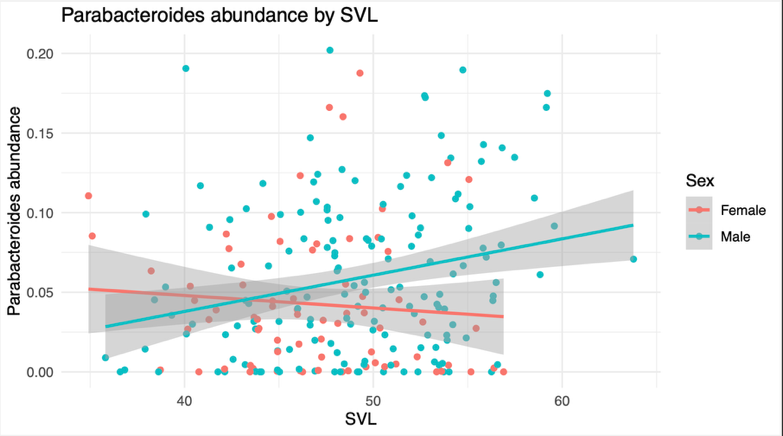

Supplement: Supplementary file 1 — Figure S1: Comparison of observed versus expected abundances in the mock microbial community after DADA2 processing. Figure S2: Canonical Analysis of Principal Coordinates (CAP) ordinations of gut microbiome composition based on weighted UniFrac and Bray‐Curtis dissimilarities. Figure S3: Gut microbiome composition comparison between male and female Podarcis lusitanicus at the phylum level. Figure S4: Relationship between snout‐vent length (SVL) and the relative abundance of Parabacteroides. Each point represents an individual lizard, with colours distinguishing different groups (e.g., sex). The blue regression line indicates a slight positive trend. The shaded area represents the confidence interval of the regression model. Table S1: Variables and corresponding values used to categorise different factors (sex and fire regime) in generalised linear model analyses. Table S2: Most abundant bacterial genera (or highest resolved taxonomic level) across all samples based on mean relative abundance. Table S3: PERMANOVA pairwise comparisons testing the effects of fire history, sex, and body size on gut microbiome beta diversity using three distance metrics (Unweighted UniFrac, Weighted UniFrac, and Bray‐Curtis). [file MEC-35-e70255-s002.docx]
